# The nuclear magneto-electric response of a chiral molecule *via* molecular dynamics in a time-dependent electric field

**DOI:** 10.1039/d5cp02294k

**Published:** 2025-10-03

**Authors:** Mateusz A. Słowiński, Juha Vaara, Piotr Garbacz

**Affiliations:** a Faculty of Chemistry, University of Warsaw, Pasteura 1 02-093 Warsaw Poland pgarbacz@uw.edu.pl; b NMR Research Unit, University of Oulu P.O. Box 3000 FI-90014 Oulu Finland

## Abstract

A chiral molecule with a permanent electric dipole moment aligns partially in an external electric field, preventing antisymmetric nuclear spin interactions from averaging out. Molecular dynamics simulations were used to investigate two such interactions – antisymmetric nuclear magnetic shielding and indirect spin–spin coupling in the light fluorinated alcohol, 1,1,1-trifluoropropan-2-ol. The results show that the rate at which a radiofrequency electric field oscillates significantly influences the spin states induced by these interactions, particularly when the frequency approaches a few gigahertz. This effect can be explained by considering dielectric losses in the electromagnetic field, which alter the amplitude and phase of the chirality-sensitive signal. As a result, at sufficiently high frequencies, the signal phase associated with a specific enantiomer may become reversed.

## Abstract 2

Chirality-dependent nuclear states in liquid-phase molecules follow an external electric field analogously to their permanent electric dipole moments.

## Graphical abstract

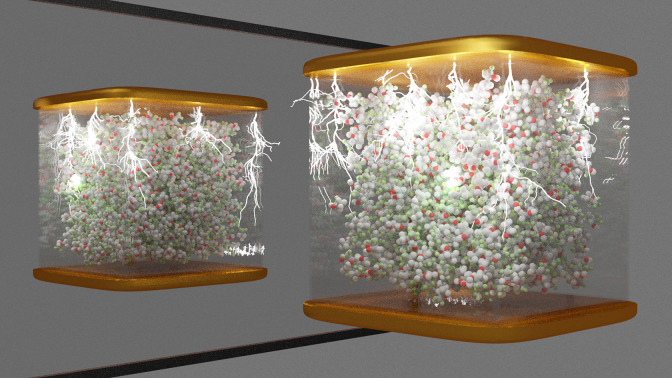

## 1. Introduction

In nuclear magnetic resonance (NMR) spectroscopy, antisymmetric nuclear interactions are closely linked to molecular chirality.^1^ Two key interactions exhibiting this antisymmetry are magnetic shielding^2^ and indirect spin–spin coupling.^3^ This study extends previous theoretical approaches used in this field by moving beyond tensor symmetry-based statistical averaging over molecular ensembles.^4^ Instead, we directly analyze detailed molecular trajectories from molecular dynamics (MD) simulations. Notably, we relax the assumption that molecules with a permanent dipole moment instantly align with an external electric field – a simplification that becomes questionable even at relatively low frequencies on the order of a fraction of a gigahertz. For instance, at room temperature, the real part of the dielectric permittivity of propan-2-ol measured at a frequency of 0.6 GHz is only one-half of that obtained for a static electric field.^5,6^ The model adopted in this work explicitly accounts for the internal friction within the fluid arising from intermolecular interactions. Considering the importance of internal molecular dynamics in NMR, this approach is essential for more realistic results, and we present an application to 1,1,1-trifluoropropan-2-ol (hereafter referred to as TFP; in the following, the results are exemplified by the (*R*)-enantiomer of TFP).

We employ three computational tools to compute the expected, chirality-dependent nuclear magnetoelectric resonance (NMER) response of a polar liquid (the summary is provided in Table S1 in the SI; tables marked with the letter S, which are referred to later in the text, can similarly be found in the SI). Initially, we used density-functional theory methods (see Section 2.1 for details) to determine the components of the antisymmetric nuclear spin interaction tensors and their dependence on the central intramolecular degree of freedom, namely the dihedral angle defined by the H–C–O–H atoms, which is involved in a large-amplitude motion in this system. Subsequently, we utilized molecular dynamics simulations to track the time evolution of the components of the local reference frame vectors of the molecule, expressed in the laboratory coordinate system. Finally, the parameters obtained from both methods were incorporated into calculations of the time-dependent amplitudes of quantum states using a master equation of the spin system in the Lindbladian form.^7–9^

In constructing the master equation of the spin system, we focused on the ^19^F and ^1^H nuclei in the TFP molecule (see Table S2 for details of the assignment of the nuclei). Specifically, we examined the fluorine nuclei in the CF_3_ group, considering their antisymmetric shielding, as well as their interactions with the CH proton, in the antisymmetric indirect spin–spin coupling. We assumed that the fluorine atoms in the CF_3_ group undergo rapid exchange (due to the rotation of the group, which is very fast in the NMR time scale) and can, therefore, be treated as equivalent. Consequently, instead of analyzing a C^19^F_3_–C^1^H four-spin system, we restricted our analysis to a ^19^F–^1^H two-spin system with magnetic shielding and spin–spin coupling tensors averaged over the fluorine nuclei. We assumed dipolar relaxation to dominate over other relaxation mechanisms. The dipolar relaxation between ^19^F nuclei and the CH proton was described using the dipolar relaxation superoperator. The relaxation terms originating from interactions between the ^19^F nuclei and between the protons of the CH_3_ group and those of the CH proton were incorporated phenomenologically as the *T*_1_ and *T*_2_ values (see Table S1 for details).

It is convenient to express the Hamiltonian 
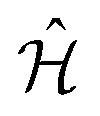
 of the ^19^F–^1^H spin system in a TFP molecule, subjected to both a magnetic field ***B*** and an electric field ***E***, using an irreducible tensor decomposition with respect to three-dimensional rotations. Specifically, the isotropic (rank-0) part of a two-index tensor 𝔗 is defined as one-third of its matrix trace, *i.e.*, 
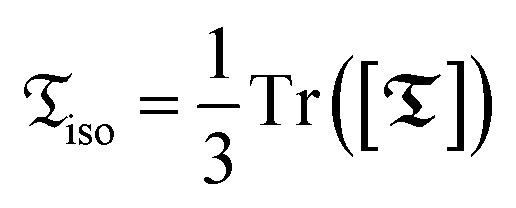
. In further text, the non-bolded symbols represent the isotropic components of the respective tensors. The star notation 
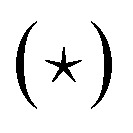
 indicates the antisymmetric (rank-1) part of a tensor, defined as half the difference between the matrix and its transpose 
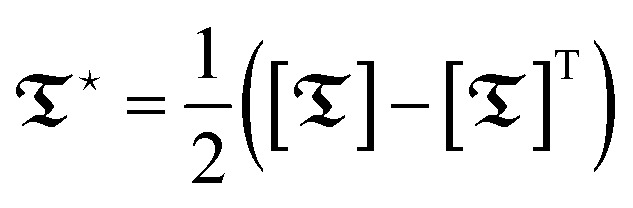
; see ref. 2 for a rigorous description. Here, the traceless symmetric (rank-2) components of the shielding tensor and the indirect spin–spin coupling tensor are neglected, as they are small for ^1^H and ^19^F in 1,1,1-trifluoropropan-2-ol, as compared to the direct coupling tensor components. By definition, the direct coupling tensor is traceless and symmetric; consequently, both its isotropic and antisymmetric components vanish.^10^

Let ^19^F be the first spin and ^1^H the second. Then, their nuclear spin Hamiltonian is given by1
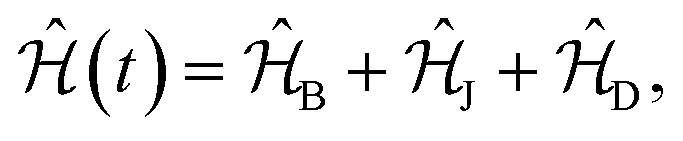
where the interaction of the nuclear spins with the ***B*** field is2
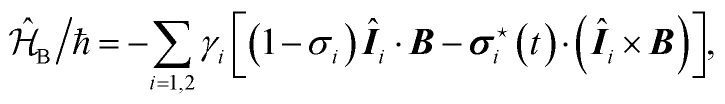
and the indirect and direct interactions between the spins are, respectively,3
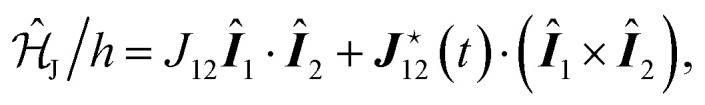
4
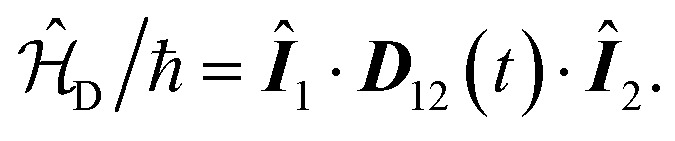


In eqn (2)–(4), ***Î***_*i*_ are dimensionless spin operators for nuclei *i* = 1, 2. The parameters *γ*_*i*_ and ***σ***_*i*_ denote the gyromagnetic ratio and the nuclear magnetic shielding tensor of nucleus *i*. The tensors ***J***_12_ and ***D***_12_ describe the indirect and direct spin–spin coupling interactions between the two nuclei.

One can analyze the electric field ***E***, the magnetic field ***B***, and the spin operators ***Î***_*i*_ in the laboratory frame, while treating the interaction tensors – ***σ***_*i*_, ***J***_12_, and ***D***_12_ – in the molecular frame.^11^ The time dependence of the antisymmetric vectors 
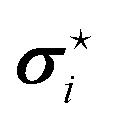
 and 
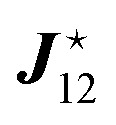
, as well as the ***D***_12_ tensor is given by5
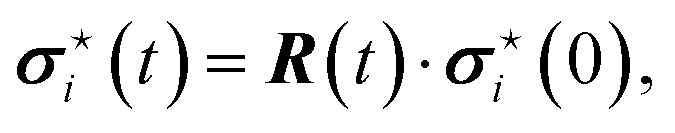
6
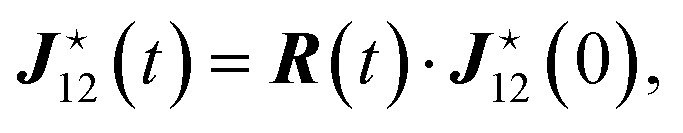

7 ***D***_12_(*t*) = ***R***(*t*)·***D***_12_(0)·***R***^T^(*t*)

where ***R***(*t*) is the proper rotation matrix that transforms the tensor components from their initial molecular orientation to their orientation at time *t*; det(***R***) = +1. Notice that the components of the antisymmetric nuclear properties (
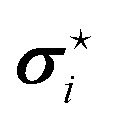
 and 
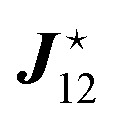
) transform as (pseudo)vectors, while the rotation of the symmetric components of the nuclear interaction tensors (*i.e.*, ***D***_12_) is governed by the formula appropriate to tensors of the second rank that are irreducible under three-dimensional rotations.^12^

The overall spin dynamics was computed by solving the master equation of the spin system8
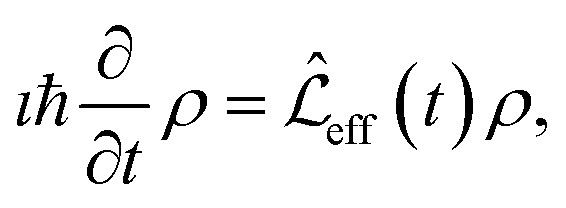
where *ρ* is the density matrix of the nuclear spin states, *ı* is the imaginary unit, and *ħ* = 1.054571817 × 10^−34^ J s is the reduced Planck's constant. The Lindbladian operator is expressed in the interaction frame as9
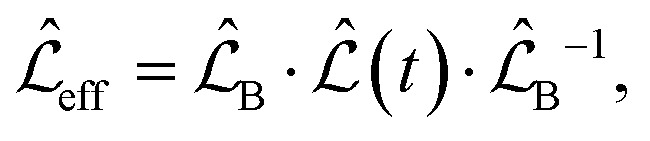
where10
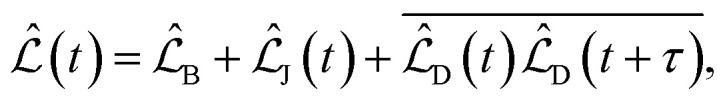
and 
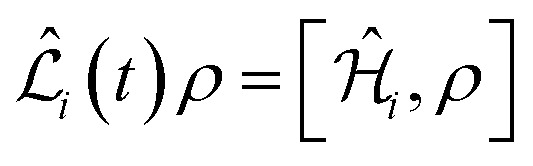
 for *i* = B, J, D. The transformation in eqn (9) takes us to the so-called rotating frame – in terms of classical physics, it corresponds to the frame rotating for each spin with its precession frequency. This transformation allows one to avoid solving the first-order differential equation system with time-dependent coefficients, given in eqn (8). Moreover, from an experimental perspective, this transformation eliminates terms that oscillate much faster than the bandwidth of a typical NMR analogue-to-digital signal conversion system at a high magnetic field.

## 2. Materials and methods

### 2.1. Quantum chemistry computations

The equilibrium geometry of TFP was optimized at the density-functional theory (DFT) level using the PBE0 exchange–correlation functional,^13^ the DFT-D3(BJ) dispersion correction^14,15^ and the def2-QZVPP basis set^16^ on the ORCA 6.0.1 programme,^17^ using the integration grid 7 (in the ORCA terminology). The optimization was started from the geometry indicated in Table S3, and the resulting atomic coordinates are given in Table S4.

After the geometry optimization, a relaxed surface scan was performed using ORCA at a similar level of theory as described above, in which the HC(OH) dihedral angle was stepped between the values of 180 and −170 degrees in steps of 10 degrees (altogether 36 different values), and all the other structural parameters were relaxed for each value of the dihedral angle. The ^3^***J***(^19^F,^1^H) spin–spin coupling tensors for the three distinct fluorine centres (F_a_, F_b_ and F_c_) of the CF_3_ group, as well as the fluorine shielding tensors ***σ***(^19^F) for the same nuclei, were calculated using the Turbomole programme^18^ using DFT, the PBE0 functional and integration grid 7 (in the Turbomole terminology), as well as the scalar-relativistic X2C level of theory^19^ and the x2c-QZVPPall-s basis set.^20^

When reporting non-scalar molecular properties, such as the NMR tensors or the electric dipole moment vector in this work, one has to specify the used molecule-fixed coordinate frame. This is particularly so when different values of the intramolecular coordinates are used, such as in the case where flexible molecular models are subjected to molecular dynamics simulations^21^ or, as in the present work, where we want to investigate the anisotropic molecular properties as a function of the HC(OH) dihedral angle, which undergoes large-amplitude motion in TFP. To this end, the calculated properties were transformed, for each relaxed geometry appropriate to the fixed HC(OH) dihedral angle, to the Eckart frame determined by the optimized equilibrium geometry (Table S4) of TFP, omitting the O(H) proton in the transformation. The method and software used in ref. 21 were employed.

### 2.2. Molecular dynamics

The structure of the lowest-energy (*R*)-conformer, obtained from DFT computations (Table S5), was used as the initial structure for the TFP molecules. The initial configuration for the MD simulations consisted of a 4.93 × 4.93 × 4.93 nm box containing 828 TFP molecules, prepared using the PACKMOL package.^22^ The molecular density used in the simulations corresponds to the experimental liquid density of TFP at room temperature, as reported in ref. 23, *ρ*_TFP_ = 1.26 g cm^−3^.

The energy of this molecular ensemble was minimized using GROMACS 2024.5^24–26^ with the OPLS-AA force field,^27,28^ employing the parameters obtained from the LigParGen web server,^29^ which are similar to those described in ref. 30–33. Force field parameters used in our study are listed in Table S6 in the SI and give the permanent electric dipole moment of TFP that reasonably agrees with that obtained from quantum chemistry calculations (see Section 3.3 and Fig. 4 for details).

First, the energy was minimized over approximately 50 000 steps, with an initial force tolerance of 12 kJ mol^−1^ nm^−1^ and a step size of 0.01 nm, followed by a second minimization step with a force tolerance of 10 kJ mol^−1^ nm^−1^ using the Verlet steepest descent algorithm. Subsequently, the system was equilibrated for 300 ps with the constant number of molecules, volume, and temperature (NVT) ensemble at *T* = 298 K, with periodic boundary conditions and temperature coupling set to 1 ps. The final equilibration step was performed with the constant number of molecules, pressure, and temperature (NPT) ensemble, where the pressure was maintained at approximately *p* = 1 bar, using the isotropic Parrinello–Rahman pressure coupling method with a coupling constant of 3.0 ps (fast as compared to the inverse of the largest used frequency, *i.e.*, 100 ps), and the temperature was kept at *T* = 298.15 K by using the Nose–Hoover algorithm. The compressibility of the system was set to 1.332 GPa^−1^, corresponding to propan-2-ol, the most similar liquid for which experimental data were available.^34^

The molecular trajectory computations were performed in GROMACS 2021.2,^35^ where the external electric field was implemented following the procedure described in ref. 36. We performed the computations both without the electric field and for the amplitude of the electric field ranging from 10^−3^ to 10 V nm^−1^. The field frequency varied from 0.03 GHz to 10 GHz and oscillated with a cosine time dependence; see Table S7 for further details.

### 2.3. Spin dynamics

The computations of the changes in time of the spin states were performed using add-on Spin Dynamica 3.0.1^37^ in the Mathematica 11 package.^38^ The effective Lindbladian operator was calculated analytically. The trajectories of the spin-state amplitudes were computed numerically based on the interpolated dependence of the averaged molecular parameters, 
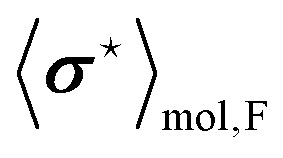
 and 
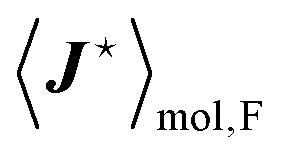
, on the frequency of the electric field, ***E***(*t*) = *E*_0_ cos(*ω*_E_*t*)***ê***_E_ with *E*_0_ = 1 V nm^−1^. The main magnetic field was assumed to be ***B***_0_ = *B*_0_***ê***_*Z*_ with *B*_0_ = 11.75 T. The density matrix of the ^19^F–^1^H spin system in the thermodynamic equilibrium (*T* = 300 K) was used as the initial mixed spin state. In the studies of antisymmetric magnetic shielding, the pulse sequence consisted only of a single excitation by the electric field of the rectangular envelope, ***E***(*t*) = *E*_0_ cos(*ω*_F_*t*)***ê***_*X*_, where *ω*_F_ is the spin precession frequency in the field ***B***_0_ (approx. 470 MHz). However, in the case of the antisymmetric indirect spin–spin coupling, first an RF-pulse was used to selectively invert the proton spin state (180° pulse), and then the rectangular pulse of the electric field was applied as ***E***(*t*) = *E*_0_ cos((*ω*_H_ − *ω*_F_)*t*)***ê***_*Z*_, where *ω*_H_ is the spin precession frequency of the proton. The chirality-sensitive states were expressed in the case of the antisymmetric magnetic shielding using the Cartesian product operators, while for the antisymmetric spin–spin coupling, single-transition operators, described in detail in ref. 39 and 40, were used.

## 3. Results

### 3.1. Probability distribution of TFP rotamers

**Fig. 1:**
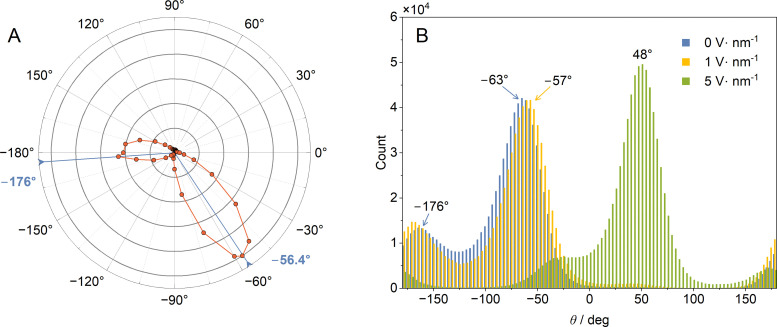
(A) Boltzmann probability distribution of the HC(OH) dihedral angle derived from DFT computations. The distance from the origin is proportional to the Boltzmann probability at temperature *T* = 300 K of the existence of the conformer with the indicated dihedral angle, with the energy obtained from the DFT calculations. (B) The variation of the dihedral HC(OH) angle (*θ*) distribution of TFP as a function of the electric-field strength obtained from MD simulations.

Chirality-sensitive NMER effects are determined by the permanent electric dipole moment of the TFP molecule, ***μ***^e^, and the antisymmetric nuclear properties (
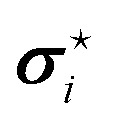
 and 
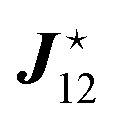
) which, in turn, are dependent on the molecular conformation; so we started our studies by finding the probability distribution of TFP rotamers. Quantum chemistry calculations indicate that TFP has two low-energy conformers that differ in the orientation of the hydroxyl group. The variation of the molecular energy with this angle is given in Table S8 of the SI. In the lowest-energy conformer, the HC(OH) dihedral angle is −56.4°, while for the second-lowest-energy conformer, this angle is −176.0° (Fig. 1A). From DFT computations it follows that, at 300 K, the ratio of the number of molecules adopting the HC(OH) angle within ±30° of, on the one hand, the first and, on the other hand, the second lowest-in-energy conformers, is approximately 3 : 1. Molecular dynamics simulations reproduce this result well (Fig. 1B). The lowest-energy rotamer structures agree with those obtained in the infrared/Raman^41,42^ and microwave^43^ studies of TFP.

### Dependence of 
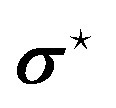
 and 
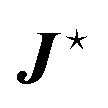
 on the TFP conformation

3.2.

Although one might suspect that the permanent electric dipole moment ***μ***^e^ should be treated as a global property of the molecule, and the antisymmetric properties (
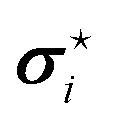
 and 
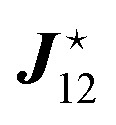
), are mainly determined by the local electronic environment of the nuclei, we have performed systematic calculations of the HC(OH) dihedral angle dependencies of all the three quantities. Such dependences were used further in computing time-variation of ***μ***^e^, 
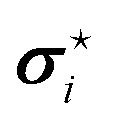
 and 
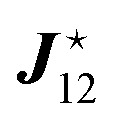
, based on the results of MD simulations.

**Fig. 2:**
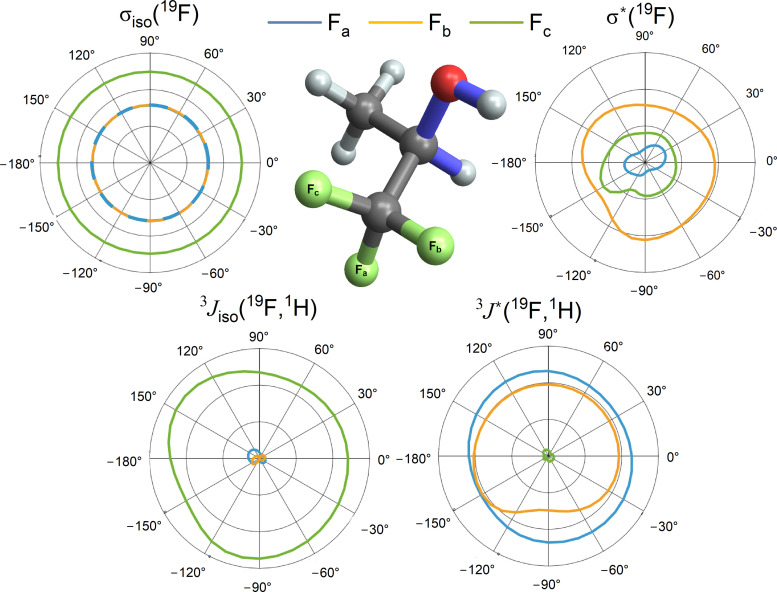
The structure of the lowest-energy conformer of TFP obtained from DFT computations (in the middle of the figure). The Corey–Pauling–Koltun atom colouring was used: hydrogen – white, carbon – black, oxygen – red, fluorine – green. The bonds forming the HC(OH) dihedral angle, *θ* = −56.4°, are highlighted in blue. Angular dependence of the isotropic and antisymmetric components of the tensors (circular diagrams): fluorine magnetic shielding, ***σ***(^19^F), and fluorine–proton indirect coupling, ^3^***J***(^19^F,^1^H) as functions of the HC(OH) dihedral angle. One radial division on the plots represents 200 ppm for *σ*_iso_(^19^F), 4 ppm for 
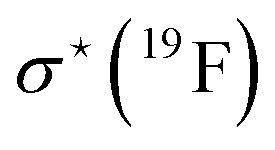
, 10 Hz for ^3^*J*_iso_(^19^F,^1^H), and 1 Hz for 
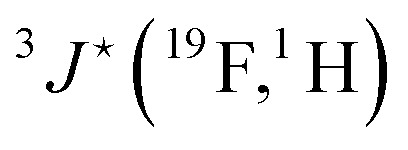
.

The isotropic parts of the magnetic shielding for the CF_3_ fluorine nuclei, *σ*_iso_(^19^F_*i*_), where *i* = a, b, c, have roughly comparable magnitudes and they almost do not vary with the HC(OH) angle (Fig. 2). A similar relationship holds for antisymmetric components, 
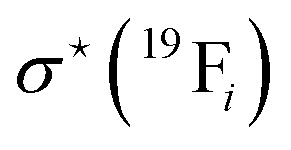
, but the relationship of 
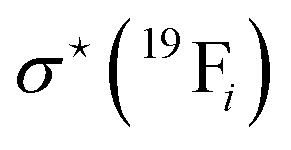
 on the HC(OH) angle is more visible than for the case of the isotropic contributions. Their Cartesian components are given in Table S9 in the SI. In contrast, the ***J***_12_ tensor components vary significantly between the fluorine nucleus involved, ^19^F_a_, ^19^F_b_, or ^19^F_c_. The isotropic part of the indirect spin–spin coupling tensor between ^19^F_c_ and the CH proton, ^3^*J*_iso_(^19^F_c_,^1^H), is noticeably larger than the corresponding couplings ^3^*J*_iso_(^19^F_a_,^1^H) and ^3^*J*_iso_(^19^F_b_,^1^H). However, the opposite is observed for the antisymmetric component of the indirect spin–spin coupling: the coupling to ^19^F_c_ is negligible compared to 
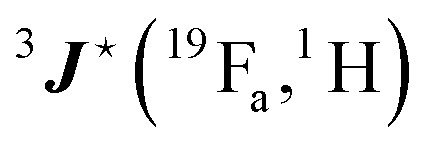
 and 
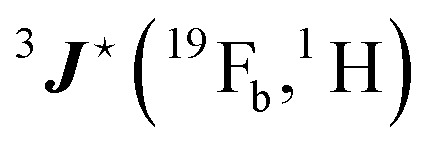
; see Table S10 in the SI for all their components.

**Fig. 3:**
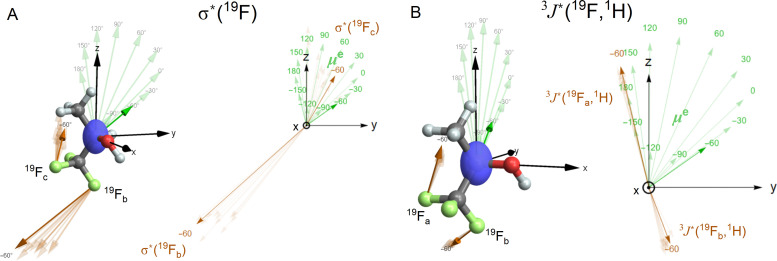
Three-dimensional view and, in the *yz*-plane, the projection of the permanent electric dipole moment (***μ***^e^, green arrow) and (A) the magnetic shielding 
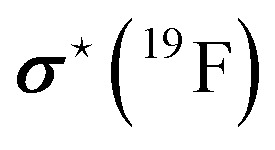
 for nuclei F_b_ and F_c_ and (B) the antisymmetric components of the indirect spin–spin coupling 
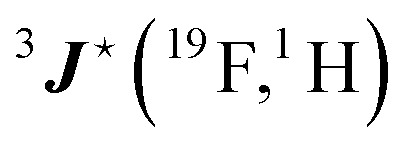
 (orange arrows) for nuclei F_a_ and F_b_. The numbers indicate the value of the HC(OH) dihedral angle. The vectors corresponding to the conformer closest to the most stable conformation are boldfaced, with HC(OH) approximately equal to −60°. We show in the blue colour the ellipsoid spanned by eigenvectors of lengths that are proportional to the absolute values of the eigenvalues of the tensor 
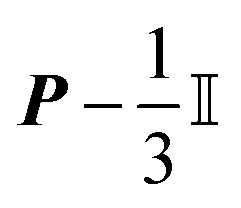
, where 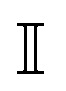 is the unity matrix.

Although the isotropic and antisymmetric components of both the magnetic shielding tensor and the indirect ^19^F–^1^H coupling tensor vary with the rotation of the hydroxyl group, these variations are relatively small in the context of the present study. Instead, the quantity that is quite sensitive to the hydroxyl group rotation angle, is the permanent electric dipole moment of the TFP molecule, ***μ***^e^ (Fig. 3).

### 3.3. Dependence of the averaged ***μ***^e^**of TFP on the electric-field strength**

To find the optimal electric-field strength, *i.e.*, such that its effect on the reorientational dynamics of TFP exceeds the stochastic noise but is sufficiently low to make reasonably accurate predictions for the experimentally accessible electric-field strengths (approximately up to 0.01 V nm^−1^), we performed MD simulations of the partial orientation of the TFP molecules using the static electric field.

If the sample is partially oriented by an externally applied static electric field, ***E*** = *E*_0_***ê***_E_, the total dipole moment averaged over the molecules follows the Langevin equation,11
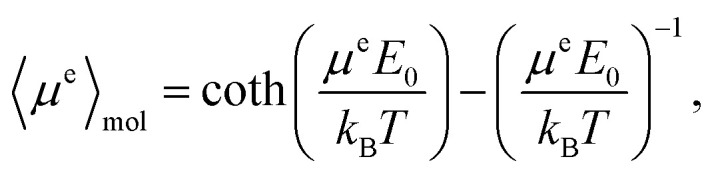
where *k*_B_ = 1.380649 × 10^−23^ J K^−1^ is the Boltzmann constant and *T* is the temperature, the bracket 〈⋯〉_mol_ means an average over molecules. For small electric fields, such that 
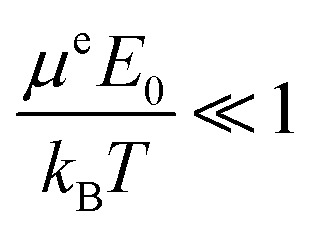
, the Langevin equation simplifies to12
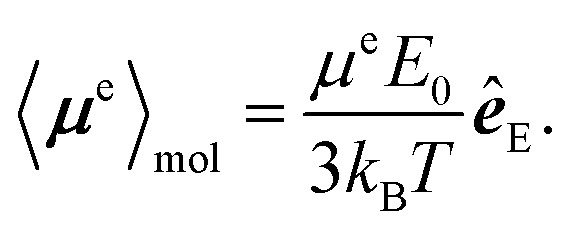


If the molecules were spherical, to describe the ordering effect of the electric field, it would be enough to calculate the average dipole electric moment from eqn (11). However, it is not evident to which extent this assumption is met by the TFP molecule due to its non-spherical shape and its internal motion. To more fully describe the field-ordering influence, the orientational probability tensor

13 ***P*** = 〈***ê***_*i*1_ ⊗ ***ê***_*i*2_〉_mol_,

should be considered. In eqn (13), the symbol ⊗ denotes the outer product, *i.e.*, (***ê***_*i*1_ ⊗ ***ê***_*i*2_)_*j*1,*j*2_ = (***ê***_*i*1_)_*j*1_(***ê***_*i*2_)_*j*2_. The unit vector ***ê***_*i*_ of the *xyz*-molecular frame (with *i* = *x*, *y*, *z*) components (***ê***_*i*_)_*j*_ is expressed in the *XYZ*-laboratory frame (with *j* = *X*, *Y*, *Z*). We chose the molecular frame of the 1,1,1-trifluoropropan-2-ol molecule as follows. The ***ê***_*x*_ vector is from the oxygen to the central carbon atom, the ***ê***_*y*_ vector is perpendicular to the ***ê***_*x*_ vector and in the plane spanned by the O–CH fragment of the molecule, and the ***ê***_*z*_ vector is perpendicular to both the ***ê***_*x*_ and ***ê***_*y*_ vectors. With the static electric field strength 1 V nm^−1^, the eigenvalues of the tensor ***P*** derived from the MD simulations are 0.35, 0.35 and 0.30. Thus, they do not significantly deviate from the isotropic rotational diffusion for which one expects all eigenvalues to equal one third. Consequently, we can consider the influence of an electric field of strength up to 1 V nm^−1^ as a small perturbation of the rotational dynamics of TFP.

**Fig. 4:**
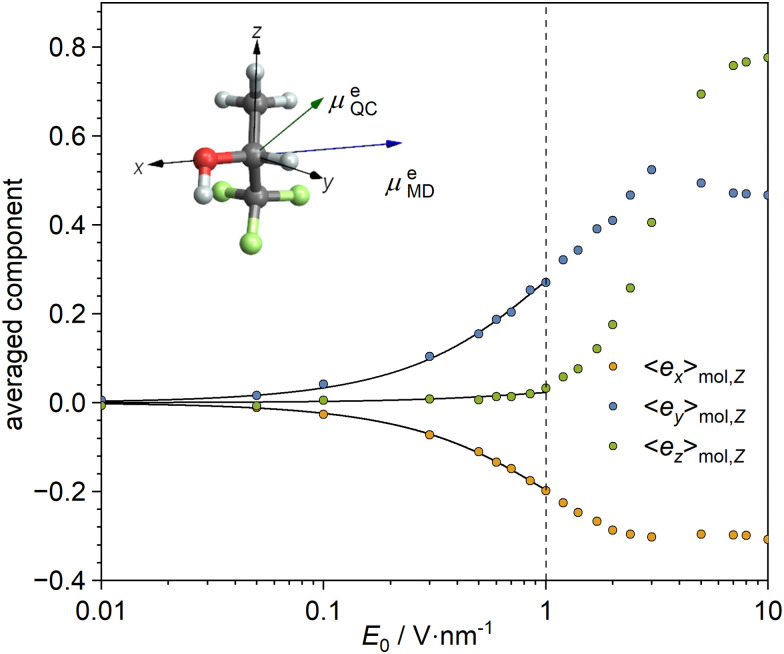
The components of the ensemble-averaged unit-vector components (〈*ê*_*i*_〉_mol,*j*_) of the TFP molecular frame vectors (〈***ê***_*i*_〉_mol_) when subjected to a static electric field ***E***_0_ aligned along the *Z*-axis of the laboratory frame that were obtained from MD (points). The solid lines represent the best fits to the Langevin function, *α*_L_(coth(*β*_L_*γ*_L_*E*_0_) − (*β*_L_*γ*_L_*E*_0_)^−1^), with parameters: *α*_L,*X*_ = −0.375(28) for 〈*ê*_*x*_〉_mol,*Z*_, *α*_L,*Y*_ = −0.525(38) for 〈*ê*_*y*_〉_mol,*Z*_, and *α*_L,*Z*_ = 0.044(6) for 〈*ê*_*z*_〉_mol,*Z*_. The used value of the parameter *β*_L_ = 0.811 D^−1^ V^−1^ nm corresponds to the temperature *T* = 300 K. The parameter *γ*_L_ = 2.37(22) D was fitted globally to all data sets above. The left inset depicts the TFP molecule with the permanent electric dipole moment used in molecular dynamics (***μ***^e^_MD_) and that obtained from quantum chemistry calculations (***μ***^e^_QC_).

The dependence of the ensemble-averaged molecular-frame axis components, 〈*ê*_*i*_〉_mol,*j*_, on the strength of the electric field obtained from MD, is shown in Fig. 4 and agrees with eqn (11). Although the orientations of the electric dipole moment of the TFP molecule at the equilibrium geometry, as derived from quantum chemistry calculations and MD differ to some degree, by approximately 30° (see the inset in Fig. 4), to report results consistently for molecular quantities derived from the same kind of computations, we decided to use the ***μ***^e^ derived from quantum chemistry. For the electric field strength lower than *E*_0_ = 0.1 V nm^−1^, the degree of orientation was smaller than the amplitude of the fluctuations. On the other hand, a larger electric-field strength, *i.e.*, *E*_0_ > 10 V nm^−1^, eventually causes saturation of the electric polarization of the sample. Moreover, at such high electric fields, most TFP molecules adopt an HC(OH) angle close to +60°, which is not the most stable conformation at lower field strengths (see Fig. 1B and Fig. S1). Therefore, for further studies, we decided to adopt a constant strength of the electric field equal to *E*_0_ = 1 V nm^−1^ (*i.e.*, 
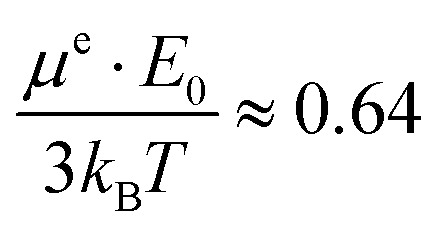
), which still corresponds to a linear dependence of the averaged moment 〈***μ***^e^〉_mol_ on the electric field strength, *i.e.*, it follows eqn (12); see Fig. S2 for the linear approximation compared the components 〈*ê*_*i*_〉_mol,*j*_ obtained from MD simulations.

### 3.4. Dependence of the averaged ***μ***^e^**of TFP on electric-field frequency**

Next, we used an electric field of the optimal strength, *i.e.*, 1 V nm^−1^, which was found in Section 3.3, and studied the average response of ***μ***^e^ of the TFP molecules as a function of the frequency of such a field, utilizing the conformational dependence determined in Section 3.2.

**Fig. 5:**
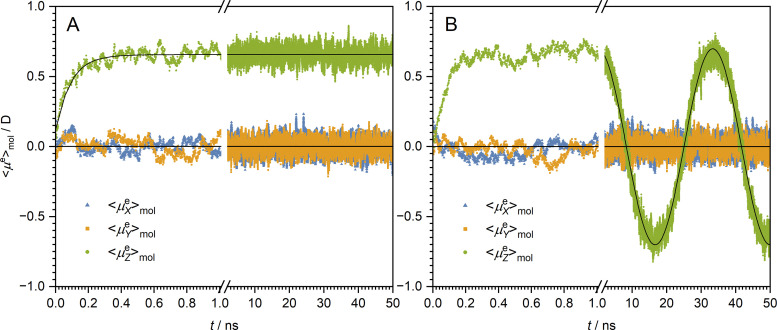
The evolution of the laboratory-frame components of the averaged electric dipole moment of TFP molecules, 〈***μ***^e^〉_mol_, under an external electric field of 1 V nm^−1^ along the *Z*-axis: (A) response to a static field ***E*** and (B) oscillating field with the frequency *f* = 0.03 GHz. The points are data obtained from MD, and the lines are the best fits for exponential decay and cosine function, respectively.

If the electric field is oscillating in time with the frequency *ω*_E_,

14 ***E***(*t*) = *E*_0_ cos(*ω*_E_*t*)***ê***_E_,

then the electric dipole moment does not follow the electric field instantaneously. Instead, the dipole moment oscillations are retarded compared to the electric field. Consequently, in the Debye model, the dipole averaged over molecules, 〈***μ***^e^〉_mol_, becomes^44^15
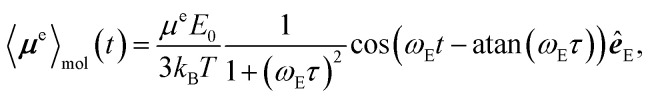
where *τ* is the diffusion reorientation time. For times sufficient to partially orient the electric field (approximately 1 ns, see Fig. 5), eqn (15) reproduces well the trends observed in MD studies. The rotational diffusion reorientation time of TFP obtained from the MD studies is *τ* = 94(2) ps.

Applying the Fourier transform to eqn (15) one obtains the frequency dependence of 〈***μ***^e^〉_mol_ in a more compact form,16
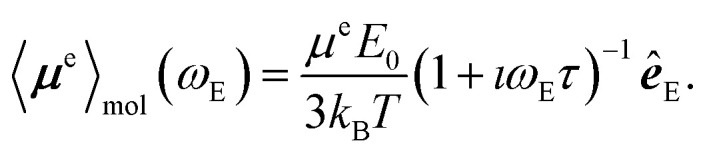


**Fig. 6:**
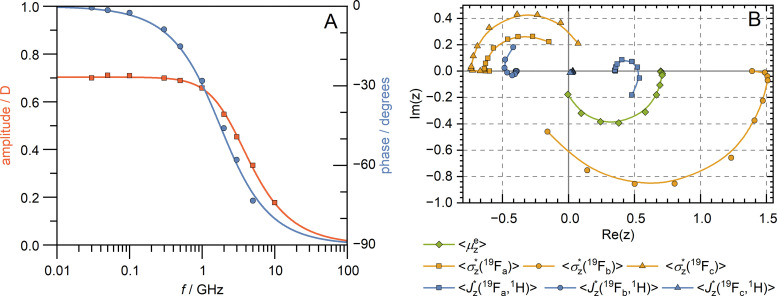
(A) Dependence of the amplitude and phase shift of the *Z*-component of the averaged dipole moment, 〈*μ*^e^_*Z*_〉_mol_, on the electric-field frequency. (B) Dependence of the averaged antisymmetric magnetic shielding, 
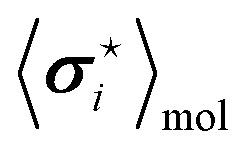
, and indirect spin–spin coupling, 
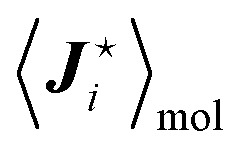
, involving fluorine nuclei ^19^F_*i*_ (*i* = a, b, c) in the complex plane on the electric-field frequency. In panel (B), the amplitude of a given nuclear property corresponds to the distance from the origin, while the phase shift is the angle relative to the positive real axis.

The dependencies of the amplitude and phase of the *Z*-component of the averaged dipole moment, 〈*μ*^e^_*Z*_〉_mol_, on the electric field frequency are shown in Fig. 6A, while the plot of the real and imaginary parts of the moment 〈***μ***^e^_*Z*_〉_mol_ is in Fig. 6B – the green curve.

### Dependence of the averaged 
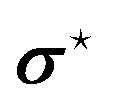
 and 
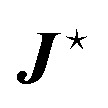
 on electric-field frequency

3.5.

To find out how the antisymmetric 
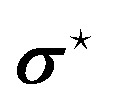
 and 
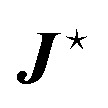
 depend on the electric-field frequency, we combined the results presented in Section 3.2 with the MD simulations at optimal ***E***-field strength (see Section 3.3) at various frequencies of the ***E***-field. Similarly to the dipole moment ***μ***^e^, the antisymmetric 
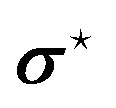
 and 
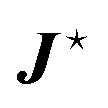
 are vector quantities placed in the same molecular frame. Hence, we assumed that they depend on the electric-field frequency analogously as found for the averaged ***μ***^e^ of TFP. The results given in Section 3.5 provide frequency dependencies used further as input data in the spin dynamics computations described in Section 3.6.

The dependence of the antisymmetric magnetic shielding averaged over molecules, 
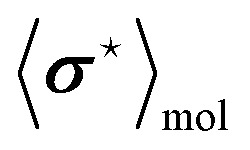
, on the frequency of electric field can be found by projecting the vector 
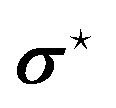
 onto the vector ***μ***^e^. The electric field orients the permanent electric dipole moment of the molecule, so the scalar projection 
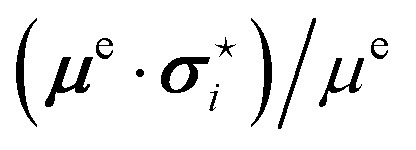
, is along the unit vector ***ê***_E_. Therefore, taking into account that the averaging over the molecules introduces a factor 
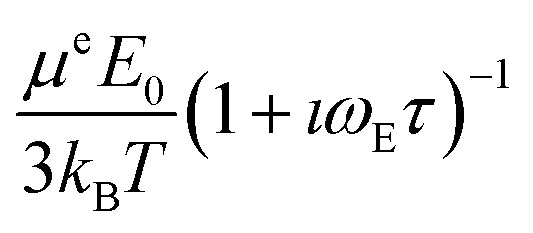
, one finds that17
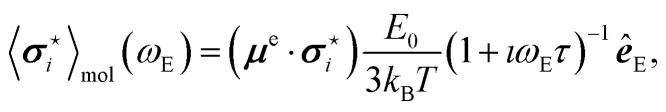


The analogous reasoning applies to the indirect spin–spin coupling 
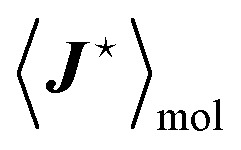
 and it gives18
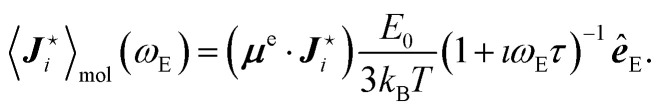


The frequency dependencies of the molecular parameters 
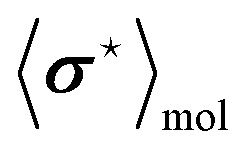
 and 
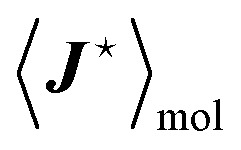
 for each fluorine nuclei are shown in Fig. 6B.

In general, eqn (17) and (18) reproduce the dependencies of the *Z*-components of antisymmetric vectors 
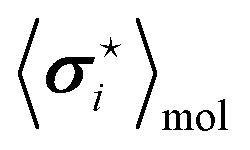
 and 
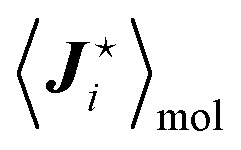
 on the frequency of the electric field *ω*_E_. However, these properties do not vanish at high electric field frequencies, as expected based on eqn (17) and (18). Such a discrepancy is an artefact of the MD simulation rather than a result of some major deviations from the assumptions of the Debye model (*e.g.*, the absence of intermolecular interactions). Although one has to admit that such assumptions are not fully satisfied in the case of TFP, since the dihedral angle HC(OH) varies by approximately 15° with electric field oscillations (Fig. S3). Consequently, the vector ***μ***^e^, onto which the antisymmetric vectors 
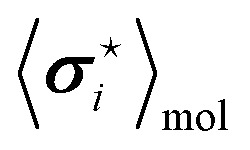
 and 
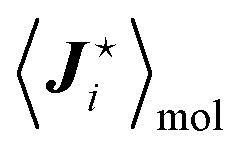
 are projected, follows the electric field in a nontrivial way. It should also be noted that in the case of molecular dynamics simulations that use the electric field directly,^45^ obtaining accurate results is a challenge compared to the approach using the fluctuation–dissipation theorem.^46,47^ Moreover, the localized charge of the CF_3_ group, which significantly contributes to the dielectric dynamics of TFP, is a source of further uncertainties in the performed MD simulations, possibly requiring the refinement of force field parameters.

### 3.6. Dependence of chirality-sensitive observables on electric-field frequency

To find the frequency dependence of the amplitudes and phases of the chirality-sensitive observables, we used the formulae 
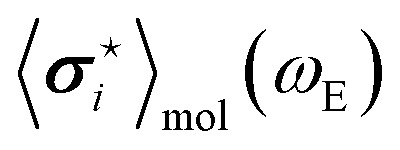
 and 
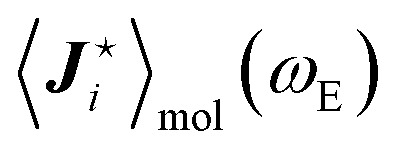
 given in eqn (17) and (18). The results were used as parameters in the spin dynamics computations of the ^1^H–^19^F system, described in the Introduction.

In the fast exchange regime, one observes an average over the fluorine nuclei,19
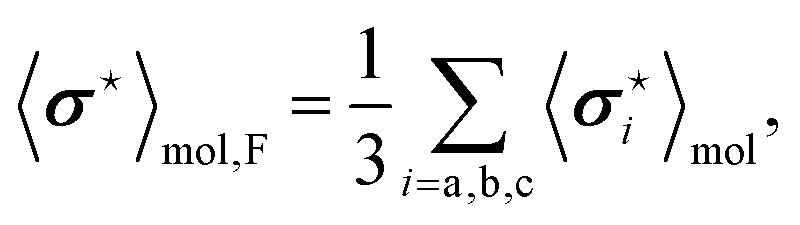
20
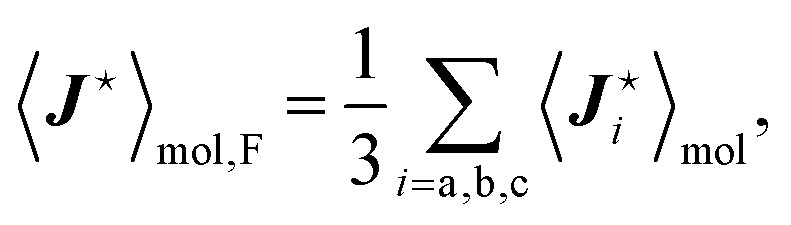


**Fig. 7:**
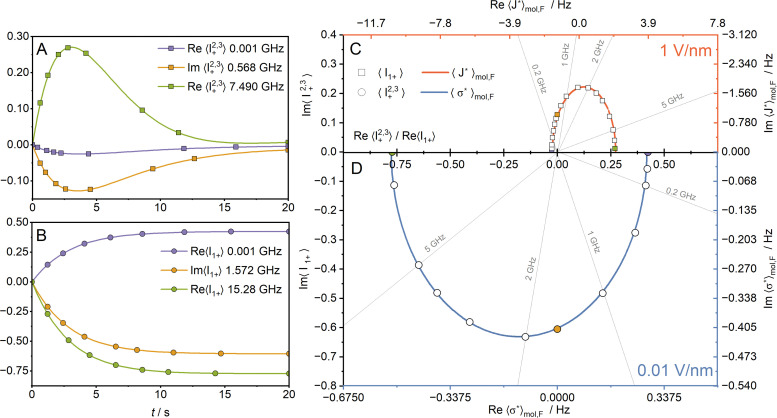
Time dependence of selected quantum state amplitudes under an oscillating electric field applied at the frequency corresponding to the difference between the proton and fluorine spin precession frequencies (A), and the fluorine spin precession frequency (B), *B*_0_ = 11.75 T, *i.e.*, 1 ppm corresponds to approximately 470 Hz, compared with the variation of the corresponding antisymmetric indirect spin–spin coupling, 
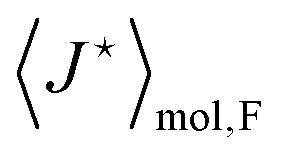
 and magnetic shielding, 
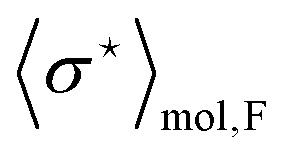
. These values were averaged over the three fluorine nuclei. The amplitudes of 〈*Î*_+_^2,3^〉 and 〈*Î*_1+_〉 after application of the 180° pulse on the proton followed by the electric-field pulse *E*_0_ cos((*ω*_H_ − *ω*_F_)*t*)***ê***_*Z*_ (C) and the electric-field pulse *E*_0_ cos(*ω*_F_*t*)***ê***_*X*_ (D), respectively. Both coherence amplitudes were normalized dividing by a factor 
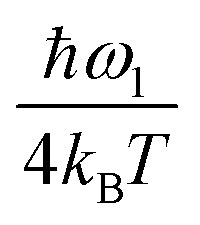
 that corresponds to the amplitude of the coherence 〈*Î*_1*x*_〉 obtained by exciting the sample at thermodynamic equilibrium at *T* = 300 K by using a 90° radiofrequency pulse.

According to Fig. 6B, the contributions of the nuclei F_a_ and F_c_ to 
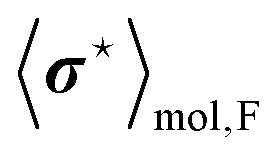
 partially cancel out with the contribution of the nucleus F_b_. For 
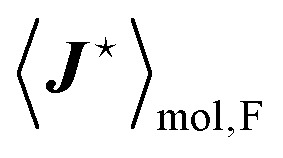
, the contributions F_a_ and F_b_ partially average out with each other. The resulting frequency dependencies of 
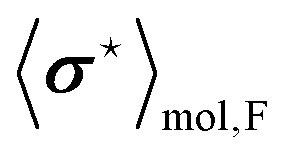
 and 
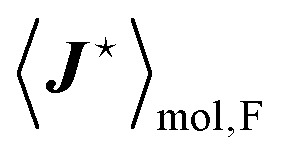
 are shown in Fig. 7.

If the first nucleus is fluorine and the second the proton, the application of the electric field oscillating at the frequency equal to the difference between the proton and fluorine spin precession frequencies, *ω*_H_ − *ω*_F_, results in the generation of chirality-sensitive spin states given by a single-transition operator *Î*_+_^2,3^ = *Î*_*X*_^2,3^ + *ıû*_*Y*_^2,3^ that are dependent on the averaged antisymmetry 
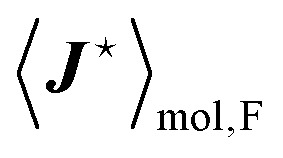
.^48^ The indices of single-transition operators denote the state |↑_F_↓_H_〉 for “2” and the state |↓_F_↑_H_〉 for “3”. In terms of the Cartesian product operators, one finds that Re(*Î*_+_^2,3^) = *Î*_1*X*_*Î*_2*X*_ + *Î*_1*Y*_*Î*_2*Y*_ and Im(*Î*_+_^2,3^) = *Î*_1*X*_*Î*_2*Y*_ − *Î*_1*Y*_*Î*_2*X*_. The comparison, shown in Fig. 7C, between amplitude-phase dependence of 
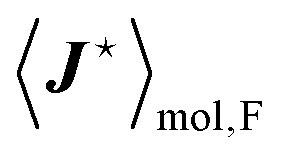
 on the frequency of the electric field (the red curve) and the computed amplitudes of the states (open squares) indicates the frequency dependence of the amplitude of the induced chirality-sensitive spin state by the electric field ***E***21
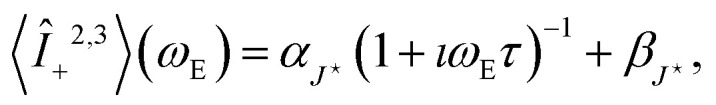
where the 
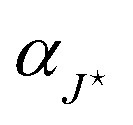
 and 
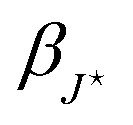
 constants account for averaging over the equivalent fluorine nuclei.

Application of the electric field oscillating at the fluorine spin precession frequency, *ω*_F_, yields chirality-sensitive spin states *Î*_1*X*_ and *Î*_1*Y*_ whose amplitudes are dependent on the averaged antisymmetry 
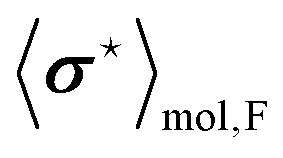
 shown in Fig. 6B. From the point of view of the spin dynamics studies, an electric field of strength 1 V nm^−1^ is so strong that it could potentially saturate nuclear magnetisation, which is unrealistic, taking into account the experimentally available electric-field strengths (in practice, lower than several kV mm^−1^ due to electric breakdown). Therefore, we took advantage of the linear dependence, for *E*_0_ < 1 V nm^−1^, of the liquid response on the field strength and scaled down the 
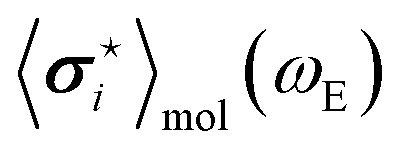
 dependence by two orders of magnitude. Analogously to the case of the antisymmetric spin–spin coupling, comparing the amplitude-phase dependence of 
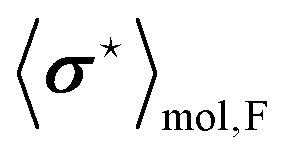
 on the frequency of the electric field (the blue curve) with the computed amplitudes of the states (open circles), one can see a close correspondence up to a multiplication by a constant (Fig. 7D). Therefore, the frequency dependence of the amplitude of the chirality-sensitive spin state *Î*_1+_ = *Î*_1*X*_ + *ıû*_1*Y*_ is22
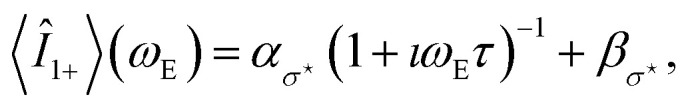
with constants 
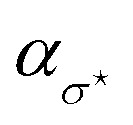
 and 
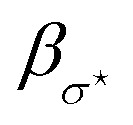
. If the discrepancies between the Debye model and the MD simulation results were to be attributed to errors that accumulate during the averaging of fluorine nuclei, then the MD data should be shifted so that 
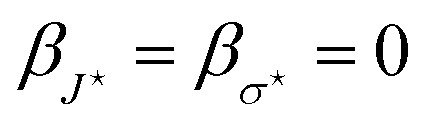
.

## 4. Discussion and conclusions

The absolute configuration of the molecule primarily governs the phase of the chirality-sensitive NMER signal. The quantities that are influenced by the configuration of the molecule are the pseudovectors of 
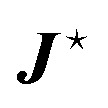
 and 
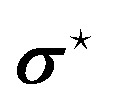
, whose directions are reversed under the transformation of one enantiomer into the other. The largest effects are those where the magnitude of the pseudovector is large. However, the frequency of the externally applied electric field also plays a significant role in modulating this phase. At sufficiently high frequencies, the signal phase may deviate considerably from its limiting behaviour observed at low frequencies, where the electric field varies slowly compared to the timescale of molecular rotational diffusion (Fig. 7A and B).

This frequency-dependent behaviour appears to be an intrinsic feature of chirality-sensitive measurements performed at high magnetic fields. Due to the inherently small magnitude of NMER signals induced by time-dependent electric fields, enhancing the initial magnetization through electric field application becomes a practical necessity for detection. Consequently, the observed signal inevitably reflects the dynamic interplay between molecular motion and the electric-field frequency.

Although the frequency dependence may seem complex, it can be quantitatively described by considering how the key parameters of the NMER effect – namely, the antisymmetric component of the nuclear magnetic shielding tensor and the antisymmetric part of the indirect spin–spin coupling – respond to the applied field frequency. Both quantities, which are vector-like, exhibit frequency dependencies analogous to that of the permanent electric dipole moment of the molecule, see eqn (16)*vs.*eqn (17) and (18).

By selecting suitable observables – such as the amplitude of the raising operator 〈*Î*_1+_〉 in the case of antisymmetric shielding, and the amplitude of the single-transition operator 〈*Î*_1+_^2,3^〉 in the case of antisymmetric spin–spin coupling – one can establish a direct correspondence between the frequency-dependent retardation of the dipole moment and the resulting phase shift in the chirality-sensitive NMER signal (eqn (21) and (22); Fig. 7C and D). This approach is particularly relevant in the radiofrequency range typically employed in NMR experiments, extending up to slightly above 1 GHz. According to the obtained results in our study, the frequency at which the signal phase associated with a specific TFP enantiomer may become reversed is approximately 2 GHz, and it corresponds to the highest point of the red curve in Fig. 7C for 
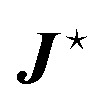
-dependent NMER effect and the lowest point of the blue curve in Fig. 7D for the 
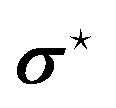
-dependent NMER effect. Moreover, the analysis of small, rigid molecules reveals that a tempting assumption – that such molecules reorient rapidly enough for their dipole moments to follow the electric field instantaneously – is not universally valid. This condition may not be satisfied even for molecules with molecular masses around 100 g mol^−1^.

The impact of the present analysis on both experimental design and expected observables depends critically on the frequency of the applied electric field, which is set chiefly by the spin system considered. For experiments with quasi-static or slowly varying fields (ref. 48), the applicability is limited. Conversely, in W-band EPR (∼100 GHz; ref. 11), the outcome is governed by the extent to which the dipole moment can follow the field.

Rather than a limitation, this sensitivity to reorientational dynamics can be viewed as a valuable source of information. Specifically, the frequency dependence of the phase shift in the NMER signal offers a novel means of probing the rotational mobility of chiral molecules in solution. Furthermore, the detection of chirality-sensitive effects mediated by antisymmetric spin–spin coupling in solution provides a compelling alternative to solid-state approaches, where such effects manifest as subtle perturbations of the spectral lineshapes *via* the parameter 
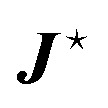
 – a measurement that is experimentally very demanding.^49,50^

These results underscore the value of integrating quantum chemistry, molecular dynamics and spin dynamics to capture chirality-sensitive nuclear interactions under realistic conditions. By explicitly accounting for finite molecular reorientation times, this framework offers a refined understanding of NMER effects and points toward new spectroscopic strategies for studying molecular motion and chirality in solution.

## Conflicts of interest

There are no conflicts to declare.

## Supplementary Material

CP-027-D5CP02294K-s001

CP-027-D5CP02294K-s002

## Data Availability

The data supporting this article have been included as part of the SI: (1) Three Wolfram Mathematica 11 files used for computing the amplitudes of the states 〈*Î*_1+_〉 (anti-s(19F)_TFP.nb) and 〈*Î*_+_^2,3^〉 (anti-3J(19F,1H)_TFP.nb), and processing data from molecular dynamics (Supplementary_Information.nb); (2) thirteen Mathematica packages used as auxiliary functions described in detail in Table S11; (3) ten text files with molecular parameters (nuclear interaction tensors and permanent electric dipole moment of TFP) described in detail in Table S12, (4) the Origin 9.60 file containing the MD ensemble-averaged components of the TFP molecular frame vectors and the best fits of Langevin curves to them. The Mathematica files can be accessed using the free Wolfram Player software, available for download at https://www.wolfram.com. See DOI: https://doi.org/10.1039/d5cp02294k. Source data for this article, including results of molecular dynamics simulations performed in Gromacs are available at RepOD at https://doi.org/10.58132/CQYOLA.
