# Supplementary material for: The nuclear magneto-electric response of a chiral molecule via molecular dynamics in a time-dependent electric field
Source: Phys Chem Chem Phys. 2025 Oct 3;27(42):22343–53. doi: 10.1039/d5cp02294k (PMC12495393; doi:10.1039/d5cp02294k)
Supplement: CP-027-D5CP02294K-s001 [file CP-027-D5CP02294K-s001.pdf]

Supplementary Information (SI) for Physical Chemistry Chemical Physics.
This journal is © the Owner Societies 2025

Nuclear Magneto-Electric Response of a Chiral Molecule via Molecular Dynamics in a Time-Dependent Electric Field

Mateusz Słowiński[1], Juha Vaara[2], Piotr Garbacz[1]

[1]Faculty of Chemistry, University of Warsaw, ul. Pasteura 1, 02-093, Warsaw, Poland

[2]NMR Research Unit, University of Oulu, P.O. Box 3000, FI-90014 Oulu, Finland

Supplementary Information

Table of contents

Tables

Table S1. Summary of the computational tools and the workflow of the conducted computations. 2

Table S2. The assignment of the nuclei of the 1,1,1-trifluoropropan-2-ol (TFP) molecule. 3

Table S3. The Z-matrix of the TFP molecules used as an initial geometry for optimization.* 4

Table S4. The Cartesian coordinates of the nuclei of the lowest-energy conformer of TFP obtained from quantum chemistry computations.* 5

Table S5. The Cartesian coordinates of the nuclei of the lowest-energy conformer of TFP obtained from quantum chemistry computations given in the *molecular* frame.* 5

Table S6. Molecular force-field parameters of TFP.* 6

Table S7. List of molecular dynamics data for TFP.* 7

Table S8. Energies the conformers of TFP given relative to the energy of the lowest one.* 8

Table S9. $\sigma(^{19}F)$ magnetic shielding tensors of TFP in the Eckart frame defined by the optimized geometry in Tab. S4 for selected dihedral angles. 9

Table S10. $^{3}J(^{19}F, ^{1}H)$ coupling tensors of TFP in the Eckart frame defined by the optimized geometry in Tab. S4 for selected dihedral angles. 12

Table S11. Auxiliary Mathematica scripts used for processing of TFP data*. 15

Table S12. Files required for Mathematica file Supplementary_Information.nb[1]. 17

Figures

Figure S1. The mean value of the dihedral angle HC(OH) 18

Figure S2. The laboratory-frame components of the ensemble-averaged unit vectors of the TFP molecular frame 19

Figure S3. The time-dependence of the mean value of the dihedral angle HC(OH) 20

Supplementary information of the article

Nuclear Magneto-Electric Response of a Chiral Molecule via Molecular Dynamics in a Time-Dependent Electric Field

Mateusz Słowiński, Juha Vaara, Piotr Garbacz

Table S1. Summary of the computational tools and the workflow of the conducted computations.

Computation type and the used software	Quantum Chemistry (ORCA and Turbomole)	Molecular Dynamics (Gromacs)	Spin Dynamics (SpinDynamica)
Input	Cartesian coordinates of atoms forming the TFP molecule	Optimal geometry of the lowest-energy conformer of TFP derived from DFT	$\langle \boldsymbol{\mu}^{\mathrm{e}}(E_0)\rangle_{\mathrm{mol}}$, $\langle \boldsymbol{\sigma}^{\star}(^{19}\mathrm{F},\ ^{1}\mathrm{H})(E_0, \omega_{\mathrm{E}})\rangle_{\mathrm{mol}}$, $\langle\ ^{3}\boldsymbol{J}^{\star}(^{19}\mathrm{F},\ ^{1}\mathrm{H})(E_0, \omega_{\mathrm{E}})\rangle_{\mathrm{F,mol}}$ $\langle \sigma_{\mathrm{iso}}(^{19}\mathrm{F})\rangle_{\mathrm{mol}}$, $\langle\ ^{3}J_{\mathrm{iso}}(^{19}\mathrm{F})\rangle_{\mathrm{mol}}$
Computed quantities	$\boldsymbol{\mu}^{\mathrm{e}}(\theta)$, $\boldsymbol{\sigma}^{\star}(^{19}\mathrm{F})(\theta)$, $^{3}\boldsymbol{J}^{\star}(^{19}\mathrm{F},\ ^{1}\mathrm{H})(\theta)$, $\sigma_{\mathrm{iso}}(^{19}\mathrm{F})(\theta)$, $^{3}J_{\mathrm{iso}}(^{19}\mathrm{F})(\theta)$	$\boldsymbol{R}(t)$, $\theta(t)$ of TFP molecules subjected to the electric field	$\langle \hat{I}_{1+}\rangle(\omega_{\mathrm{E}})$ for $\boldsymbol{\sigma}^{\star}$ $\langle \hat{I}_{+}^{2,3}\rangle(\omega_{\mathrm{E}})$ for $\boldsymbol{J}^{\star}$
Constraints / assumptions	DFT-optimized geometry with a series of fixed dihedral angles θ	NPT ensemble (N=828, p=1 bar, T=300 K); electric field is either constant or $\boldsymbol{E}(t) = E_0 \cos(\omega_{\mathrm{E}} t)\, \hat{\boldsymbol{e}}_z$	$^{1}\mathrm{H}$-$^{19}\mathrm{F}$ two-spin system with phenomenological relaxation due to CF_3 and CH_3 groups: $T_1(\ ^{1}\mathrm{H}) = T_2(\ ^{1}\mathrm{H}) = \left(3 b_{\mathrm{HH}}^{2} \tau_2\right)^{-1}$ $T_1(^{19}\mathrm{F}) = T_2(^{19}\mathrm{F}) = \left(3 b_{\mathrm{FF}}^{2} \tau_2\right)^{-1}$, where the distances for the dipolar constants are $r_{\mathrm{HH}} = 2.67$ Å (mean distance between protons CH and CH_3) and $r_{\mathrm{FF}} = 2.16$ Å (mean distance between the fluorine nuclei in the CF_3 group). Fast exchange and extreme narrowing were assumed.
Derived quantities	Interpolated dependences of the computed quantities on the dihedral angle value	Probability tensor, $\langle \boldsymbol{\mu}^{\mathrm{e}}(E_0)\rangle_{\mathrm{mol}}$, $\langle \boldsymbol{\sigma}^{\star}(^{19}\mathrm{F},\ ^{1}\mathrm{H})(E_0, \omega_{\mathrm{E}})\rangle_{\mathrm{mol}}$, $\langle\ ^{3}\boldsymbol{J}^{\star}(^{19}\mathrm{F},\ ^{1}\mathrm{H})(E_0, \omega_{\mathrm{E}})\rangle_{\mathrm{mol}}$ $\langle \sigma_{\mathrm{iso}}(^{19}\mathrm{F})\rangle_{\mathrm{mol}}$, $\langle\ ^{3}J_{\mathrm{iso}}(^{19}\mathrm{F})\rangle_{\mathrm{mol}}$	N/A

Table S2. The assignment of the nuclei of the 1,1,1-trifluoropropan-2-ol (TFP) molecule.

no.	nucleus	position*
1	H	$\mathrm{C(\underline{\mathbf{H}})\text{-}OH}$
2	C	$\mathrm{\underline{\mathbf{C}}(H)\text{-}OH}$
3	O	$\mathrm{C(H)\text{-}\underline{\mathbf{O}}H}$
4	H	$\mathrm{C(H)\text{-}O\underline{\mathbf{H}}}$
5	C	$\mathrm{\underline{\mathbf{C}}H_3}$
6	H	$\mathrm{C\underline{\mathbf{H}}_3}$ (−179.8°)
7	H	$\mathrm{C\underline{\mathbf{H}}_3}$ (61.1°)
8	H	$\mathrm{C\underline{\mathbf{H}}_3}$ (−58.8°)
9	C	$\mathrm{\underline{\mathbf{C}}F_3}$
10	F	$\mathrm{C\underline{\mathbf{F}}_3}$ (53.8°); F_a
11	F	$\mathrm{C\underline{\mathbf{F}}_3}$ (−64.9°); F_b
12	F	$\mathrm{C\underline{\mathbf{F}}_3}$ (175.1°); F_c

* The dihedral angle is given in parentheses. The atoms chosen for calculation of the dihedral angles for the CH_3 and CF_3 groups are $\mathrm{\underline{\mathbf{HC}}(OH)\text{-}\underline{\mathbf{C}}H_2\text{-}\underline{\mathbf{H}}}$ and $\mathrm{\underline{\mathbf{HC}}(OH)\text{-}\underline{\mathbf{C}}F_2\text{-}\underline{\mathbf{F}}}$, respectively.

Mateusz Słowiński, Juha Vaara, Piotr Garbacz

Table S3. The Z-matrix of the TFP molecules used as an initial geometry for optimization.*

nucleus	no.	distance / Å	no.	planar angle / degrees	no.	dihedral angles / degrees
H						
C	1	1.09				
O	2	1.41	1	110.9		
H	3	0.96	2	110.9	1	120
C	2	1.50	3	110.9	1	120
H	5	1.09	2	110.9	1	−175
H	5	1.09	2	110.9	1	65
H	5	1.09	2	110.9	1	−55
C	2	1.50	3	110.9	1	−115
F	9	1.34	2	110.9	1	60
F	9	1.34	2	110.9	1	−60
F	9	1.34	2	110.9	1	180

* The optimized coordinates are given in Tab. S4.

Table S4. The Cartesian coordinates of the nuclei of the lowest-energy conformer of TFP obtained from quantum chemistry computations.*

no.	nucleus	X / Å	Y / Å	Z / Å
1	H	−0.073425	−0.104939	−0.020096
2	C	−1.165319	0.007376	−0.001615
3	O	−1.806776	−1.239009	0.002652
4	H	−1.517693	−1.745293	−0.759020
5	C	−1.587028	0.765788	1.232536
6	H	−2.670045	0.881296	1.250524
7	H	−1.282668	0.206951	2.115725
8	H	−1.122154	1.750179	1.262090
9	C	−1.509579	0.770522	−1.275430
10	F_a	−0.805588	1.904868	−1.360481
11	F_b	−1.206943	0.030314	−2.357587
12	F_c	−2.800971	1.084647	−1.362205

* This equilibrium geometry, excluding the O(H) group proton, defines the *Eckart* frame in which the calculated anisotropic molecular properties are presented.

Table S5. The Cartesian coordinates of the nuclei of the lowest-energy conformer of TFP obtained from quantum chemistry computations given in the *molecular* frame.*

no.	nucleus	X / Å	Y / Å	Z / Å
1	H	−0.39985	1.02240	0.00000
2	C	0.00000	0.00000	0.00000
3	O	1.40177	0.00000	0.00000
4	H	1.71733	0.50153	−0.75428
5	C	−0.47761	−0.74278	1.22324
6	H	−0.08466	−1.75875	1.22288
7	H	−0.11731	−0.23140	2.11410
8	H	−1.56552	−0.78045	1.25548
9	C	−0.52489	−0.63375	−1.28311
10	F_a	−1.85591	−0.52552	−1.36229
11	F_b	−0.00852	−0.00772	−2.35642
12	F_c	−0.21352	−1.92407	−1.39263

* The molecular frame is chosen such that the $\hat{\boldsymbol{e}}_x$ vector is from the oxygen to central carbon atom, the $\hat{\boldsymbol{e}}_y$ vector is perpendicular the $\hat{\boldsymbol{e}}_x$ vector and it lies the plane spanned by the O-CH fragment of the molecule, and the $\hat{\boldsymbol{e}}_z$ vector is perpendicular to the $\hat{\boldsymbol{e}}_x$ and $\hat{\boldsymbol{e}}_y$ vectors. This is the same geometry as in Tab. S4. The permanent electric dipole moment obtained from DFT computations is 1.66 D.

Mateusz Słowiński, Juha Vaara, Piotr Garbacz

Table S6. Molecular force-field parameters of TFP.*

no.	atom	position	σ / Å	ε / kJ·mol^{-1}	q/e	m/u
00	C	**<u>C</u>**H3	0.350	0.2761	−0.2869	12.0110
01	C	**<u>C</u>**(H)-OH	0.350	0.2761	0.1023	12.0110
02	H	C(**<u>H</u>**)-OH	0.250	0.1255	0.1170	1.0080
03	O	C(H)-**<u>O</u>**H	0.312	0.7113	−0.6661	15.9990
04	C	**<u>C</u>**F	0.350	0.2761	−0.4353	12.0110
05	F	C**<u>F</u>**3 (170°); F_c	0.290	0.2514	−0.1584	18.9984
06	F	C**<u>F</u>**3 (−70°); F_b	0.290	0.2514	−0.1584	18.9984
07	F	C**<u>F</u>**3 (50°); F_a	0.290	0.2514	−0.1584	18.9984
08	H	C**<u>H</u>**3 (60°)	0.250	0.1255	0.1094	1.0080
09	H	C**<u>H</u>**3 (−60°)	0.250	0.1255	0.1094	1.0080
0A	H	C**<u>H</u>**3 (180°)	0.250	0.1255	0.1094	1.0080
0B	H	C(H)-O**<u>H</u>**	0.000	0.0000	0.4272	1.0080

* Lennard-Jones potential is parametrized by the distance at which the interatomic potential is zero (σ) and the potential energy at its minimum (ε). The charges of the atoms are expressed in multiples of the elementary charge (1 e = 1.602176634·10^{-19} C). Atomic mass is reported in unified atomic mass units (Daltons), 1 u = 1.66053906892·10^{-27} kg. The permanent electric dipole moment obtained from charges listed in Tab. S6 and atomic coordinates taken from Tab. S5 is 2.01 D. The parameters obtained from the LigParGen web server. The details of the LigParGen web server are given in L. S. Dodda, I. Cabeza de Vaca, J. Tirado-Rives and W. L. Jorgensen, *Nucleic Acids Res.* 2017, 45, W331–W336.

Mateusz Słowiński, Juha Vaara, Piotr Garbacz

Table S7. List of molecular dynamics data for TFP.*

Data set. no.	Electric field		Number of molecules	Length of the data set /ns	Sampling set /ps**
	Amplitude /V·nm^{-1}	Frequency /GHz			
1	0.000	0.00	828	1	0.1
2	0.001	0.00	828	1	0.1
3	0.002	0.00	828	1	0.1
4	0.004	0.00	828	1	0.1
5	0.005	0.00	828	1	0.1
6	0.010	0.00	828	1	0.1
7	0.050	0.00	828	1	0.1
8	0.100	0.00	828	1	0.1
9	0.300	0.00	828	1	0.1
10	0.500	0.00	828	1	0.1
11	0.600	0.00	828	1	0.1
12	0.700	0.00	828	1	0.1
13	0.850	0.00	828	1	0.1
14	1.000	0.00	828	1	0.1
15	1.200	0.00	828	1	0.1
16	1.400	0.00	828	1	0.1
17	1.700	0.00	828	1	0.1
18	2.000	0.00	828	1	0.1
19	2.400	0.00	828	1	0.1
20	3.000	0.00	828	1	0.1
21	5.000	0.00	828	1	0.1
22	7.000	0.00	828	1	0.1
23	8.000	0.00	828	1	0.1
24	10.000	0.00	828	1	0.1
25−27	1.000	0.00	828	50	1.0
28−30	1.000	0.03	828	50	1.0
31−33	1.000	0.05	828	50	1.0
34−36	1.000	0.10	828	50	1.0
37−39	1.000	0.30	828	25	1.0
40−42	1.000	0.50	828	25	1.0
43−45	1.000	1.00	828	25	1.0
46−48	1.000	2.00	828	25	1.0
49−51	1.000	3.00	828	25	1.0
52−54	1.000	5.00	828	25	1.0
55−57	1.000	10.00	828	25	1.0

* This table contains the first line of each corresponding Gromacs file.

** The time step Δt was chosen such that $\Delta t \leq 10^{-5}/f_{\mathrm{max}}$, where f_{max} is the highest frequency of the applied field.

Mateusz Słowiński, Juha Vaara, Piotr Garbacz

Table S8. Energies the conformers of TFP given relative to the energy of the lowest one.*

Dihedral angle /degrees	Energy /kJ·mol^{-1}	Dihedral angle /degrees	Energy /kJ·mol^{-1}
**180**	**2.186**	0	7.939
170	2.252	−10	6.318
160	2.998	−20	4.449
150	4.245	−30	2.600
140	5.728	−40	1.076
130	7.154	−50	0.161
120	8.274	−**60**	**0.075**
110	8.965	−70	0.921
100	9.261	−80	2.645
90	9.309	−90	4.995
80	9.286	−100	7.502
70	9.326	−110	9.524
60	9.487	−120	10.417
50	9.738	−130	9.898
40	9.979	−140	8.283
30	10.061	−150	6.217
20	9.821	−160	4.286
10	9.129	−170	2.877

* Two the lowest-energy conformers of the TFP molecules have dihedral angles equal −56.68° and −176.0° computed from the coordinates of the atoms **HC**(**OH**). The former conformer has the lowest energy, while the energy of the latter is higher by 1.949 kJ·mol^{-1} then the former one. The conformers having dihedral angles that are the closest to these two lowest-in-energy conformers are boldfaced in the table.

Table S9. $\sigma(^{19}F)$ magnetic shielding tensors of TFP in the Eckart frame defined by the optimized geometry in Tab. S4 for selected dihedral angles.

Dihedral angle/ degrees	$\sigma(^{19}F_a)$ /ppm			$\sigma(^{19}F_b)$ /ppm			$\sigma(^{19}F_c)$ /ppm		
180	271.40	36.92	−38.43	279.20	−6.06	−37.42	346.17	−18.85	15.95
	34.38	319.05	5.20	5.92	214.89	59.44	−30.19	273.71	42.47
	−41.29	1.08	198.05	−31.93	70.18	295.77	15.24	42.27	218.46
170	272.10	37.54	−38.80	279.04	−5.32	−37.30	345.60	−18.58	16.35
	35.36	319.27	5.08	6.68	215.75	59.16	−29.13	274.27	41.75
	−41.35	1.16	198.12	−31.42	70.43	295.72	16.34	40.66	217.24
160	272.58	38.04	−39.14	278.72	−4.56	−37.13	345.10	−18.49	16.42
	36.34	319.50	4.94	7.34	216.56	58.98	−28.42	274.68	41.06
	−41.45	1.29	198.19	−31.07	70.65	295.78	16.89	39.22	216.52
150	272.88	38.43	−39.44	278.31	−3.83	−36.88	344.63	−18.62	16.23
	37.29	319.73	4.75	7.97	217.19	59.04	−28.03	274.84	40.48
	−41.57	1.51	198.32	−30.77	70.87	295.83	17.04	38.06	216.18
140	272.89	38.75	−39.65	277.87	−3.08	−36.55	344.19	−18.85	15.89
	38.12	319.92	4.50	8.60	217.69	59.28	−27.81	274.84	40.08
	−41.72	1.70	198.41	−30.60	71.06	295.92	16.92	37.29	216.14
130	272.64	39.06	−39.77	277.48	−2.36	−36.19	343.69	−19.15	15.52
	38.80	320.02	4.17	9.23	218.02	59.64	−27.61	274.69	39.85
	−41.86	1.72	198.34	−30.60	71.22	296.03	16.77	36.84	216.20
120	272.22	39.40	−39.78	277.22	−1.73	−35.80	343.11	−19.46	15.26
	39.35	319.98	3.79	9.86	218.16	60.13	−27.37	274.42	39.79
	−41.95	1.52	198.02	−30.76	71.33	296.17	16.77	36.56	216.20
110	271.71	39.78	−39.66	277.13	−1.24	−35.43	342.48	−19.75	15.20
	39.73	319.79	3.37	10.41	218.05	60.65	−27.05	274.06	39.92
	−41.95	1.08	197.41	−31.06	71.35	296.23	17.04	36.38	216.09
100	271.23	40.19	−39.41	277.16	−1.01	−35.15	341.88	−19.94	15.38
	39.94	319.43	2.94	10.72	217.70	61.10	−26.64	273.65	40.21
	−41.81	0.45	196.52	−31.48	71.31	296.17	17.59	36.25	215.85
90	270.86	40.60	−39.04	277.26	−1.12	−35.08	341.39	−19.99	15.74
	39.99	318.95	2.55	10.73	217.14	61.38	−26.13	273.27	40.59
	−41.54	−0.30	195.50	−31.98	71.18	296.02	18.40	36.13	215.55
80	270.64	40.98	−38.64	277.37	−1.61	−35.33	341.05	−19.89	16.20
	39.86	318.42	2.26	10.43	216.40	61.45	−25.57	272.95	40.97
	−41.22	−1.07	194.50	−32.51	70.98	295.83	19.31	36.00	215.24
70	270.55	41.24	−38.26	277.45	−2.41	−35.93	340.91	−19.62	16.64
	39.55	317.88	2.05	9.82	215.65	61.36	−24.99	272.72	41.26
	−40.86	−1.74	193.63	−33.01	70.78	295.61	20.12	35.88	215.06

Mateusz Słowiński, Juha Vaara, Piotr Garbacz

60	270.55	41.36	−37.92	277.44	−3.43	−36.86	340.98	−19.20	16.95
	39.12	317.39	1.88	8.94	215.09	61.12	−24.42	272.59	41.34
	−40.54	−2.29	193.01	−33.41	70.59	295.43	20.71	35.79	215.08
50	270.63	41.36	−37.71	277.31	−4.49	−37.97	341.19	−18.69	17.09
	38.62	316.99	1.73	7.96	214.84	60.80	−23.94	272.53	41.17
	−40.33	−2.67	192.72	−33.68	70.46	295.34	20.98	35.73	215.29
40	270.78	41.28	−37.66	277.10	−5.45	−39.11	341.46	−18.17	17.04
	38.12	316.72	1.59	7.09	214.90	60.50	−23.60	272.54	40.81
	−40.27	−2.83	192.82	−33.80	70.44	295.37	20.93	35.70	215.64
30	270.98	41.15	−37.79	276.86	−6.12	−40.13	341.66	−17.73	16.84
	37.70	316.59	1.48	6.50	215.23	60.25	−23.45	272.63	40.35
	−40.36	−2.81	193.22	−33.75	70.57	295.49	20.60	35.73	216.08
20	271.25	40.96	−38.10	276.70	−6.43	−40.90	341.73	−17.42	16.55
	37.39	316.60	1.41	6.26	215.74	60.14	−23.55	272.76	39.88
	−40.60	−2.61	193.87	−33.58	70.86	295.69	20.14	35.77	216.52
10	271.61	40.74	−38.55	276.59	−6.42	−41.38	341.71	−17.25	16.23
	37.25	316.74	1.40	6.28	216.30	60.25	−23.82	273.00	39.60
	−40.95	−2.26	194.68	−33.34	71.34	295.90	19.62	35.87	217.00
0	272.05	40.51	−39.09	276.52	−6.14	−41.58	341.61	−17.24	15.96
	37.24	316.95	1.47	6.51	216.80	60.58	−24.21	273.36	39.55
	−41.35	−1.83	195.58	−33.10	71.95	296.08	19.03	36.06	217.50
−10	272.59	40.20	−39.67	276.63	−5.66	−41.61	341.43	−17.38	15.77
	37.30	317.21	1.60	6.86	217.16	61.10	−24.72	273.74	39.73
	−41.78	−1.34	196.50	−32.82	72.70	296.26	18.50	36.30	217.99
−20	273.26	39.83	−40.32	277.12	−5.17	−41.56	341.14	−17.66	15.67
	37.40	317.45	1.76	7.14	217.38	61.85	−25.21	274.08	40.22
	−42.16	−0.86	197.45	−32.46	73.51	296.37	18.15	36.59	218.44
−30	274.03	39.45	−40.98	278.10	−4.88	−41.54	340.80	−18.06	15.65
	37.46	317.59	1.87	7.26	217.46	62.80	−25.61	274.32	40.94
	−42.46	−0.51	198.30	−32.08	74.33	296.39	18.00	36.92	218.86
−40	274.93	39.04	−41.55	279.50	−4.94	−41.63	340.39	−18.52	15.71
	37.44	317.55	1.86	7.13	217.40	63.88	−25.92	274.38	41.76
	−42.70	−0.32	198.96	−31.70	75.15	296.33	18.02	37.24	219.18
−50	275.89	38.63	−42.02	281.24	−5.45	−41.81	339.92	−19.06	15.78
	37.28	317.29	1.71	6.72	217.30	64.98	−26.10	274.12	42.61
	−42.86	−0.31	199.44	−31.28	75.88	296.23	18.26	37.49	219.30
−60	276.86	38.28	−42.32	282.99	−6.44	−42.02	339.45	−19.68	15.84
	36.99	316.79	1.38	6.12	217.21	65.97	−26.08	273.53	43.41
	−42.91	−0.55	199.72	−30.86	76.49	296.05	18.67	37.62	219.24

−70	277.68	38.04	−42.38	284.46	−7.73	−42.09	339.09	−20.40	15.87
	36.61	316.07	0.82	5.50	217.10	66.66	−25.94	272.59	44.13
	−42.89	−0.99	199.74	−30.52	76.90	295.84	19.10	37.65	218.97
−80	278.19	37.93	−42.14	285.30	−9.18	−41.90	338.97	−21.19	15.78
	36.15	315.25	0.13	4.91	216.87	66.96	−25.81	271.38	44.65
	−42.78	−1.50	199.54	−30.34	77.08	295.57	19.40	37.60	218.56
−90	278.21	37.92	−41.55	285.29	−10.49	−41.45	339.33	−22.00	15.48
	35.68	314.51	−0.52	4.39	216.50	66.81	−25.81	270.07	44.91
	−42.52	−1.93	199.12	−30.38	76.99	295.31	19.35	37.57	218.15
−100	277.48	37.98	−40.70	284.45	−11.48	−40.84	340.49	−22.62	14.86
	35.21	314.15	−0.70	3.80	215.86	66.26	−26.23	268.91	44.90
	−42.06	−1.96	198.65	−30.81	76.53	295.17	18.57	37.79	218.03
−110	275.79	37.83	−39.84	282.82	−11.94	−40.16	342.66	−22.76	13.89
	34.68	314.40	0.01	2.97	214.80	65.53	−27.46	268.07	44.65
	−41.46	−1.37	198.50	−31.75	75.57	295.11	16.65	38.57	218.49
−120	273.48	37.04	−39.08	280.80	−11.66	−39.39	345.27	−22.40	12.95
	33.93	315.22	1.53	2.15	213.25	64.66	−29.65	267.78	44.30
	−40.91	−0.30	198.79	−32.94	74.09	295.15	13.95	40.01	219.72
−130	271.42	35.75	−38.49	279.06	−10.78	−38.62	347.16	−21.84	12.54
	33.04	316.33	3.24	1.75	211.75	63.54	−32.17	268.10	43.96
	−40.68	0.76	199.26	−33.81	72.43	295.32	11.66	41.92	221.17
−140	270.37	34.87	−38.07	278.31	−9.78	−37.99	347.88	−21.36	12.83
	32.43	317.28	4.38	2.09	211.20	62.42	−33.68	269.02	43.80
	−40.76	1.36	199.32	−33.94	71.16	295.45	10.71	43.61	221.97
−150	270.19	34.80	−37.88	278.37	−8.79	−37.65	347.88	−20.86	13.54
	32.34	317.91	4.92	2.96	211.62	61.45	−33.78	270.18	43.72
	−40.96	1.47	199.00	−33.57	70.47	295.52	11.06	44.53	221.82
−160	270.41	35.28	−37.91	278.75	−7.81	−37.52	347.51	−20.17	14.41
	32.69	318.41	5.18	3.98	212.62	60.58	−32.90	271.45	43.49
	−41.14	1.36	198.64	−33.04	70.12	295.60	12.22	44.54	220.93
−170	270.89	36.05	−38.12	279.08	−6.91	−37.47	346.90	−19.47	15.27
	33.41	318.75	5.23	5.00	213.78	59.91	−31.53	272.65	43.05
	−41.25	1.19	198.31	−32.47	70.09	295.67	13.75	43.69	219.67

Table S10. $^3J(^{19}F, ^1H)$ coupling tensors of TFP in the Eckart frame defined by the optimized geometry in Tab. S4 for selected dihedral angles.

Dihedral angle/ degrees	$^3J(^{19}F_a, ^1H)$ /Hz			$^3J(^{19}F_b, ^1H)$ /Hz			$^3J(^{19}F_c, ^1H)$ /Hz		
	−2.12	−0.95	0.44	3.25	−0.64	2.53	22.47	−6.75	10.45
180	−4.64	−1.04	0.99	1.56	−1.07	3.23	−6.75	10.16	−5.27
	−1.01	2.78	−5.86	5.22	1.10	2.76	10.37	−5.08	16.06
	−2.32	−0.92	0.45	3.16	−0.67	2.48	22.76	−7.08	10.59
170	−4.67	−1.19	0.98	1.52	−1.17	3.10	−7.02	10.88	−5.69
	−0.99	2.80	−5.98	5.13	0.98	2.27	10.56	−5.47	16.73
	−2.46	−0.89	0.44	3.12	−0.69	2.44	23.02	−7.31	10.73
160	−4.72	−1.30	0.98	1.49	−1.20	2.98	−7.20	11.46	−6.04
	−0.99	2.82	−6.06	5.05	0.89	1.90	10.73	−5.78	17.35
	−2.53	−0.88	0.42	3.08	−0.70	2.39	23.16	−7.43	10.83
150	−4.78	−1.31	0.98	1.48	−1.20	2.90	−7.29	11.81	−6.28
	−1.00	2.83	−6.07	4.98	0.83	1.59	10.86	−5.99	17.79
	−2.49	−0.88	0.40	3.03	−0.70	2.34	23.15	−7.44	10.87
140	−4.85	−1.20	0.97	1.50	−1.18	2.87	−7.27	11.89	−6.37
	−1.00	2.82	−5.99	4.91	0.81	1.32	10.89	−6.06	17.95
	−2.35	−0.89	0.38	2.95	−0.68	2.30	22.95	−7.35	10.80
130	−4.94	−0.95	0.94	1.56	−1.18	2.88	−7.17	11.70	−6.33
	−0.99	2.78	−5.83	4.85	0.85	1.08	10.81	−6.00	17.80
	−2.11	−0.92	0.38	2.85	−0.63	2.27	22.58	−7.19	10.64
120	−5.03	−0.54	0.88	1.66	−1.20	2.94	−7.00	11.32	−6.16
	−0.96	2.70	−5.60	4.79	0.94	0.83	10.63	−5.83	17.37
	−1.79	−0.98	0.40	2.72	−0.56	2.26	22.11	−7.00	10.40
110	−5.12	−0.03	0.79	1.78	−1.26	3.04	−6.82	10.86	−5.94
	−0.90	2.58	−5.31	4.76	1.08	0.61	10.38	−5.62	16.78
	−1.43	−1.06	0.43	2.60	−0.48	2.29	21.62	−6.83	10.15
100	−5.21	0.54	0.69	1.90	−1.34	3.15	−6.67	10.44	−5.72
	−0.83	2.44	−5.00	4.75	1.23	0.44	10.13	−5.42	16.17
	−1.08	−1.15	0.48	2.53	−0.40	2.35	21.19	−6.70	9.91
90	−5.28	1.06	0.59	2.01	−1.42	3.26	−6.56	10.13	−5.55
	−0.75	2.31	−4.73	4.79	1.38	0.38	9.90	−5.27	15.66
	−0.78	−1.26	0.54	2.55	−0.34	2.43	20.86	−6.62	9.72
80	−5.33	1.47	0.51	2.07	−1.47	3.36	−6.51	9.96	−5.43
	−0.69	2.20	−4.52	4.87	1.51	0.47	9.72	−5.19	15.30
	−0.56	−1.35	0.59	2.65	−0.31	2.54	20.64	−6.59	9.59
70	−5.35	1.73	0.47	2.08	−1.47	3.42	−6.50	9.93	−5.38
	−0.64	2.13	−4.40	4.99	1.60	0.73	9.60	−5.17	15.14

	−0.43	−1.41	0.63	2.83	−0.30	2.63	20.52	−6.59	9.52
60	−5.34	1.84	0.46	2.03	−1.42	3.45	−6.51	10.01	−5.38
	−0.61	2.10	−4.36	5.12	1.64	1.12	9.53	−5.20	15.15
	−0.38	−1.44	0.67	3.04	−0.33	2.71	20.49	−6.62	9.50
50	−5.30	1.82	0.48	1.94	−1.32	3.44	−6.53	10.19	−5.43
	−0.60	2.12	−4.39	5.22	1.61	1.56	9.49	−5.27	15.30
	−0.41	−1.41	0.69	3.24	−0.38	2.74	20.51	−6.64	9.52
40	−5.24	1.72	0.52	1.83	−1.22	3.38	−6.54	10.40	−5.50
	−0.60	2.16	−4.44	5.29	1.54	1.95	9.47	−5.37	15.54
	−0.49	−1.35	0.70	3.41	−0.43	2.72	20.56	−6.67	9.55
30	−5.17	1.58	0.56	1.71	−1.12	3.28	−6.54	10.62	−5.59
	−0.62	2.21	−4.51	5.31	1.42	2.19	9.46	−5.47	15.80
	−0.62	−1.25	0.71	3.51	−0.49	2.66	20.63	−6.69	9.59
20	−5.10	1.42	0.61	1.62	−1.05	3.17	−6.54	10.80	−5.67
	−0.65	2.28	−4.58	5.27	1.29	2.24	9.47	−5.56	16.05
	−0.77	−1.12	0.70	3.54	−0.53	2.56	20.70	−6.71	9.63
10	−5.03	1.27	0.67	1.56	−1.00	3.05	−6.54	10.95	−5.75
	−0.69	2.34	−4.64	5.18	1.16	2.11	9.50	−5.63	16.25
	−0.93	−0.99	0.67	3.51	−0.55	2.45	20.80	−6.74	9.70
0	−4.97	1.12	0.72	1.53	−0.98	2.94	−6.54	11.05	−5.82
	−0.73	2.40	−4.69	5.07	1.04	1.83	9.55	−5.69	16.42
	−1.09	−0.86	0.63	3.44	−0.55	2.34	20.96	−6.78	9.79
−10	−4.92	0.98	0.77	1.55	−1.00	2.85	−6.56	11.14	−5.89
	−0.79	2.46	−4.73	4.95	0.94	1.45	9.65	−5.75	16.57
	−1.23	−0.76	0.57	3.36	−0.52	2.27	21.19	−6.85	9.92
−20	−4.89	0.87	0.81	1.59	−1.06	2.78	−6.61	11.24	−5.95
	−0.84	2.50	−4.76	4.82	0.87	1.01	9.80	−5.79	16.74
	−1.32	−0.70	0.51	3.26	−0.46	2.22	21.48	−6.93	10.08
−30	−4.89	0.83	0.83	1.66	−1.16	2.72	−6.68	11.35	−6.02
	−0.89	2.51	−4.76	4.70	0.82	0.55	9.99	−5.85	16.93
	−1.35	−0.68	0.46	3.18	−0.36	2.22	21.83	−7.03	10.25
−40	−4.91	0.88	0.81	1.74	−1.30	2.68	−6.78	11.50	−6.10
	−0.91	2.50	−4.72	4.58	0.79	0.07	10.21	−5.91	17.13
	−1.30	−0.71	0.42	3.12	−0.25	2.25	22.24	−7.17	10.42
−50	−4.98	1.07	0.76	1.82	−1.47	2.65	−6.92	11.72	−6.20
	−0.90	2.44	−4.64	4.46	0.78	−0.40	10.43	−6.01	17.34
	−1.17	−0.78	0.40	3.09	−0.13	2.31	22.68	−7.35	10.57
−60	−5.07	1.41	0.65	1.87	−1.65	2.63	−7.09	12.02	−6.32
	−0.85	2.34	−4.50	4.33	0.78	−0.84	10.63	−6.13	17.54
	−0.95	−0.88	0.40	3.07	−0.03	2.38	23.12	−7.55	10.69
−70	−5.18	1.91	0.50	1.90	−1.82	2.61	−7.31	12.38	−6.45
	−0.77	2.20	−4.29	4.21	0.79	−1.20	10.79	−6.28	17.71

	−0.67	−1.00	0.43	3.06	0.03	2.46	23.52	−7.74	10.76
−80	−5.29	2.51	0.33	1.88	−1.96	2.61	−7.52	12.75	−6.57
	−0.66	2.03	−4.03	4.12	0.83	−1.42	10.90	−6.42	17.82
	−0.39	−1.12	0.47	3.06	0.05	2.55	23.85	−7.87	10.78
−90	−5.39	3.08	0.18	1.85	−2.02	2.64	−7.69	13.03	−6.63
	−0.56	1.89	−3.79	4.12	0.90	−1.43	10.94	−6.49	17.86
	−0.22	−1.24	0.52	3.09	0.00	2.67	24.05	−7.84	10.76
−100	−5.46	3.32	0.14	1.82	−1.96	2.76	−7.72	13.01	−6.54
	−0.53	1.84	−3.71	4.29	1.03	−1.05	10.91	−6.42	17.79
	−0.30	−1.35	0.53	3.21	−0.13	2.82	24.06	−7.55	10.74
−110	−5.47	2.89	0.30	1.80	−1.69	2.97	−7.50	12.47	−6.21
	−0.60	1.98	−3.98	4.66	1.22	−0.07	10.83	−6.09	17.58
	−0.60	−1.38	0.49	3.45	−0.30	2.91	23.76	−6.97	10.68
−120	−5.35	1.85	0.62	1.79	−1.22	3.25	−7.00	11.38	−5.62
	−0.79	2.26	−4.49	5.12	1.42	1.49	10.66	−5.50	17.14
	−0.93	−1.31	0.41	3.68	−0.44	2.85	23.15	−6.30	10.56
−130	−5.10	0.71	0.91	1.77	−0.73	3.47	−6.38	10.07	−4.94
	−1.01	2.54	−4.95	5.42	1.53	3.11	10.41	−4.81	16.43
	−1.18	−1.20	0.36	3.75	−0.53	2.74	22.55	−5.89	10.41
−140	−4.87	−0.01	1.04	1.74	−0.52	3.58	−5.99	9.15	−4.49
	−1.12	2.69	−5.21	5.50	1.54	3.99	10.17	−4.35	15.69
	−1.40	−1.12	0.37	3.64	−0.57	2.65	22.20	−5.84	10.31
−150	−4.74	−0.38	1.06	1.70	−0.59	3.58	−5.94	8.81	−4.38
	−1.12	2.74	−5.39	5.46	1.48	4.06	10.05	−4.24	15.24
	−1.64	−1.05	0.39	3.48	−0.59	2.60	22.11	−6.05	10.30
−160	−4.66	−0.64	1.04	1.65	−0.76	3.50	−6.13	8.95	−4.53
	−1.09	2.76	−5.55	5.39	1.37	3.73	10.07	−4.39	15.17
	−1.88	−0.99	0.42	3.34	−0.62	2.56	22.23	−6.39	10.34
−170	−4.63	−0.84	1.01	1.60	−0.94	3.38	−6.43	9.47	−4.86
	−1.04	2.76	−5.71	5.30	1.24	3.23	10.19	−4.70	15.48

Table S11. Auxiliary Mathematica scripts used for processing of TFP data*.

	Script and its arguments	Input	Output
Gromacs	DataConverter<br>[*file*, *operation*]	Accepted format of the *file* is Gromacs output file (*.gro)	If “DATA” argument is used, then the script creates a text containing processed data, *i.e.*, uses the script Gromacs2RawData for the Gromacs. The optional argument “XYZ” allows one to extract atomic coordinates of a chosen molecule using the script GromacsToXYZ; see the source code for details.
	FindLocalFrameAnd<br>DihedralAngle<br>[*atoms*]	A list of Cartesian coordinates of four *atoms*. Here, we used the proton and carbon of the methanetriyl group and then the oxygen and proton of the hydroxyl group of the TFP molecule.	The rotation matrix of the molecular frame of reference, *i.e.*, $\boldsymbol{R} = \begin{pmatrix} u_x & v_x & w_x \\ u_y & v_y & w_y \\ u_z & v_z & w_z \end{pmatrix}$, where the x−axis is a normalized to unit length vector $\boldsymbol{r}_{\mathrm{CO}}$, the y−axis is a normalized to unit length component of the vector $\boldsymbol{r}_{\mathrm{CH}}$ that is perpendicular to the vector $\boldsymbol{r}_{\mathrm{CO}}$, the z−axis is a cross product of the previous two. The vector $\boldsymbol{r}_{\mathrm{CH}}$ is from **C**H(OH) to C**H**(OH) and $\boldsymbol{r}_{\mathrm{CO}}$ is a vector from **C**H(OH) to CH(**O**H).<br><br>The dihedral angle θ is computed from arctangent of the coordinates of the point which has the ordinate $r_{\mathrm{CO}}[(\boldsymbol{r}_{\mathrm{CH}} \times \boldsymbol{r}_{\mathrm{CO}}) \cdot (\boldsymbol{r}_{\mathrm{CO}} \times \boldsymbol{r}_{\mathrm{OH}})]$ and the abscissa $\boldsymbol{r}_{\mathrm{CO}}[(\boldsymbol{r}_{\mathrm{CH}} \times \boldsymbol{r}_{\mathrm{CO}}) \times (\boldsymbol{r}_{\mathrm{CO}} \times \boldsymbol{r}_{\mathrm{OH}})]$ where $\boldsymbol{r}_{\mathrm{OH}}$ is a vector from CH(**O**H) to CH(O**H**).
	ProbabilityTensor[*file*, *n*]	*File* obtained using script DataConverter[*file*, “DATA”]	It returns the probability tensor of a set containing *n* items stored in the *file*.

Spin Dynamica	Lindbladian[$\mathcal{L}_1$, $\mathcal{L}_2$,*n*]	Liouvillians $\mathcal{L}_1$ and $\mathcal{L}_2$in the superoperator form	Computes the Lindblad operator of Liouvillians $\mathcal{L}_1(0)$ and $\mathcal{L}_2(\tau)$
	LindbladIntegrate[*expr*]	Symbolic expression *expr*	Symbolically simplifies the expression *expr* according to the Lindblad theory
	LeaveOutFastOscillatingTerms [*expr*]	Symbolic expression *expr*	Leaves out terms exp(constant x *t*) in the expression *expr*
	SchrodingerToInteractionPicture [$\mathcal{L}_1$, $\mathcal{L}_2$]	Liouvillians $\mathcal{L}_1$ and $\mathcal{L}_2$in the superoperator form	Transforms the Liouvillian $\mathcal{L}_1$ in the Schrödinger picture to the interaction picture given by the Liouvillian $\mathcal{L}_2$
	OperatorCross[*i*, *j*]	operators opI[*n*]	Computes the cross product of Cartesian operators of spins $\hat{\boldsymbol{I}}_i$ and $\hat{\boldsymbol{I}}_j$.
	UseNumericalValues [*expr*, *property*, *frequency*]	Symbolic expression *exp*<br>*property*=’shielding’ ’jcoupling’<br>*frequency* in [GHz]	Substitutes numeric values relevant to *property* in the expression *expr*
	Antisymmetry [*property*, *frequency*]	*property*=’shielding’ ’jcoupling’<br>*frequency* in [GHz]	Returns the replace rule giving the numerical value of the amplitude $\mathcal{A}$ and phase ϕ of a *property* averaged over molecules at given *frequency*

*All input and output files are text files.

Mateusz Słowiński, Juha Vaara, Piotr Garbacz

Table S12. Files required for Mathematica file Supplementary_Information.nb[1].

File no.	File name	File line structure					
1	energy.dat	θ (rad)	E (kJ·mol^{-1})				
2	TFP_lowest_energy.xyz	N (nuclei)	X (Å)	Y (Å)	Z (Å)		
3	me.dat[2]	μ_x^{e} (a.u.)	μ_y^{e} (a.u.)	μ_z^{e} (a.u.)			
4	s_iso_anti.dat	θ (deg) $\sigma_{\mathrm{iso}}({}^{19}\mathrm{F_a})$	$\sigma^\star({}^{19}\mathrm{F_a})$	$\sigma_{\mathrm{iso}}({}^{19}\mathrm{F_b})$	$\sigma^\star({}^{19}\mathrm{F_b})$	$\sigma_{\mathrm{iso}}({}^{19}\mathrm{F_c})$	$\sigma^\star({}^{19}\mathrm{F_c})$ (ppm)
5	J_iso_anti.dat	θ (deg) ${}^3J_{\mathrm{iso}}({}^{19}\mathrm{F_a}, {}^1\mathrm{H})$	${}^3J^\star({}^{19}\mathrm{F_a}, {}^1\mathrm{H})$	${}^3J_{\mathrm{iso}}({}^{19}\mathrm{F_b}, {}^1\mathrm{H})$	${}^3J^\star({}^{19}\mathrm{F_b}, {}^1\mathrm{H})$	${}^3J^\star({}^{19}\mathrm{F_c}, {}^1\mathrm{H})$	${}^3J_{\mathrm{iso}}({}^{19}\mathrm{F_c}, {}^1\mathrm{H})$ (Hz)
6	sF1.dat[3]	θ (deg)	$\sigma_x^\star({}^{19}\mathrm{F_a})$ (ppm)	$\sigma_y^\star({}^{19}\mathrm{F_a})$ (ppm)	$\sigma_z^\star({}^{19}\mathrm{F_a})$ (ppm)		
7	sF2.dat[3]	θ (deg)	$\sigma_x^\star({}^{19}\mathrm{F_b})$ (ppm)	$\sigma_y^\star({}^{19}\mathrm{F_b})$ (ppm)	$\sigma_z^\star({}^{19}\mathrm{F_b})$ (ppm)		
5	sF3.dat[3]	θ (deg)	$\sigma_x^\star({}^{19}\mathrm{F_c})$ (ppm)	$\sigma_y^\star({}^{19}\mathrm{F_c})$ (ppm)	$\sigma_z^\star({}^{19}\mathrm{F_c})$ (ppm)		
6	JFH1.dat[3]	θ (deg)	${}^3J_x^\star({}^{19}\mathrm{F_a}, {}^1\mathrm{H})$ (Hz)	${}^3J_y^\star({}^{19}\mathrm{F_a}, {}^1\mathrm{H})$ (Hz)	${}^3J_z^\star({}^{19}\mathrm{F_a}, {}^1\mathrm{H})$ (Hz)		
7	JFH2.dat[3]	θ (deg)	${}^3J_x^\star({}^{19}\mathrm{F_b}, {}^1\mathrm{H})$ (Hz)	${}^3J_y^\star({}^{19}\mathrm{F_b}, {}^1\mathrm{H})$ (Hz)	${}^3J_z^\star({}^{19}\mathrm{F_b}, {}^1\mathrm{H})$ (Hz)		
8	JFH3.dat[3]	θ (deg)	${}^3J_x^\star({}^{19}\mathrm{F_c}, {}^1\mathrm{H})$ (Hz)	${}^3J_y^\star({}^{19}\mathrm{F_c}, {}^1\mathrm{H})$ (Hz)	${}^3J_z^\star({}^{19}\mathrm{F_c}, {}^1\mathrm{H})$ (Hz)		

[1] Keep them in the same directory as the Mathematica file.

[2] 1 a.u. electric dipole moment (ea_0) = 8.4783536·10^{-30} C·m ≈ 2.5417 D.

[3] Cartesian coordinates are given in the Eckart frame.

Mateusz Słowiński, Juha Vaara, Piotr Garbacz

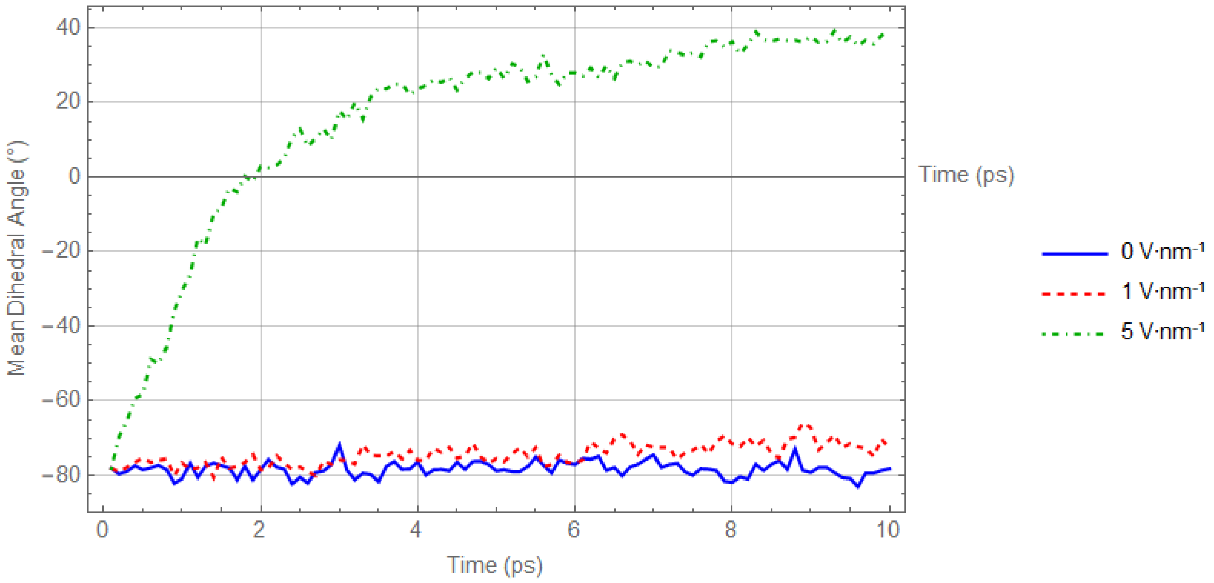

Figure S1. The mean value of the dihedral angle HC(OH) averaged over 828 TFP molecules for the first 10 ps of the molecular dynamics simulation after turning on the static electric field.

Mateusz Słowiński, Juha Vaara, Piotr Garbacz

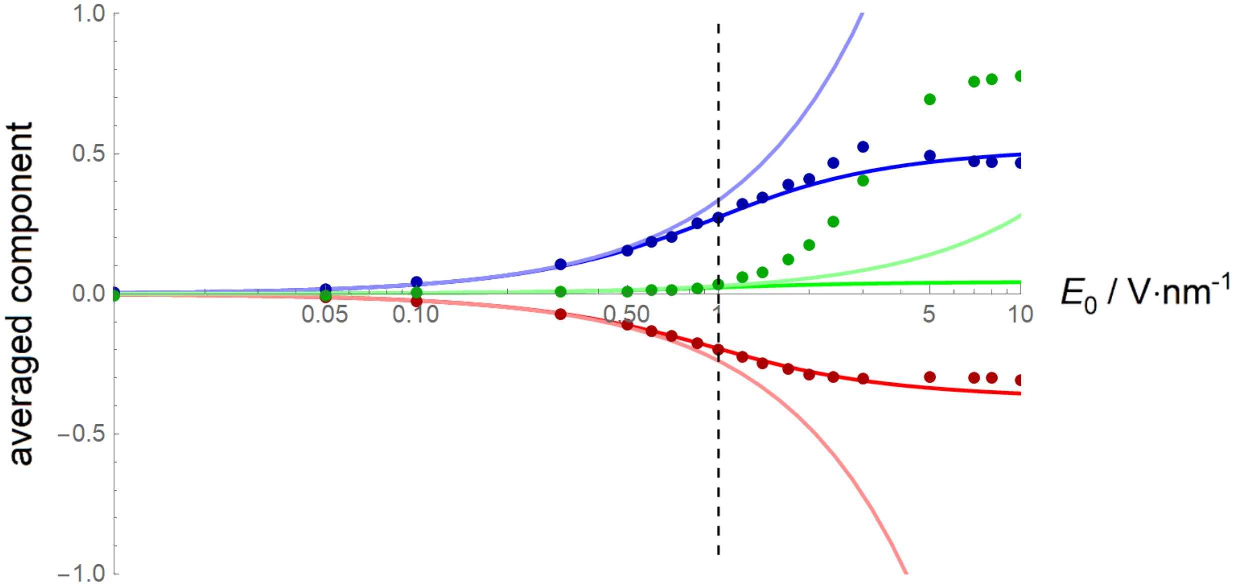

Figure S2. The laboratory-frame components of the ensemble-averaged unit vectors of the TFP molecular frame: $\langle \hat{e}_x \rangle_{\mathrm{mol},Z}$ (red), $\langle \hat{e}_y \rangle_{\mathrm{mol},Z}$ (green), and $\langle \hat{e}_z \rangle_{\mathrm{mol},Z}$ (blue). The lines in darker color are the fits using the Langevin function, $(\coth(x) - (x)^{-1})$, while the lighter lines are the low-electric field approximations, $x/3$; parameters of the functions are omitted here for clarity. See details in the main text, especially the caption of Fig. 4.

Mateusz Słowiński, Juha Vaara, Piotr Garbacz

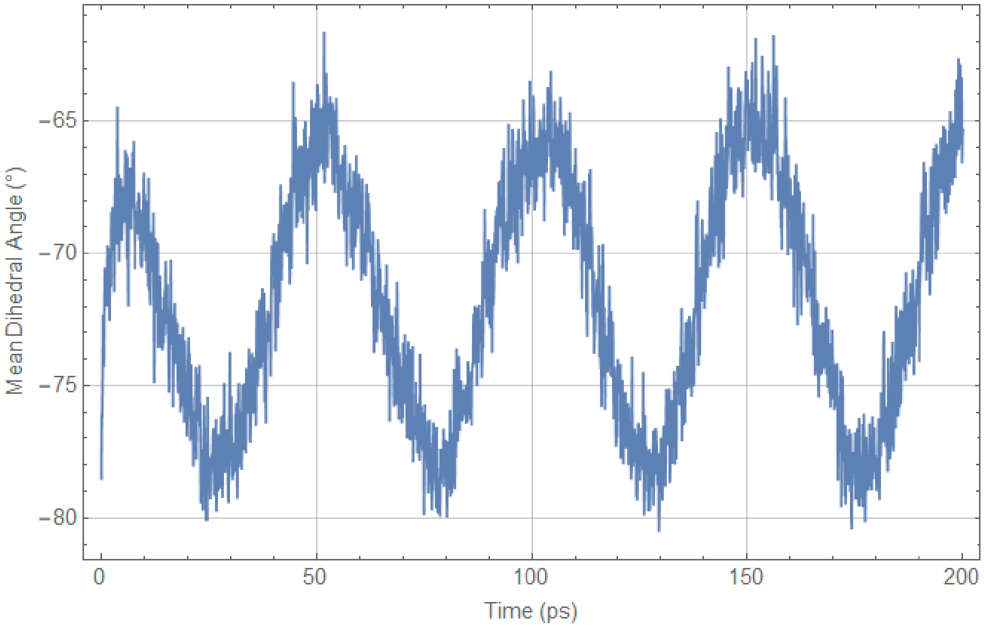

Figure S3. The time-dependence of the mean value of the dihedral angle HC(OH) averaged over 828 TFP molecules subjected to the electric field of the amplitude 1 V·nm^{-1} and frequency 1 GHz.
